# Supplementary material for: Effectiveness of seasonal influenza vaccination in patients with diabetes: protocol for a nested case–control study
Source: BMJ Open. 2017 Aug 18;7(8):e016023. doi: 10.1136/bmjopen-2017-016023 (PMC5629692; doi:10.1136/bmjopen-2017-016023)
Supplement: Author's manuscript [file bmjopen-2017-016023.draft_revisions.pdf]

BMJ Open

Effectiveness of seasonal influenza vaccination in patients with diabetes: protocol for a nested case-control study

Journal:	*BMJ Open*
Manuscript ID	bmjopen-2017-016023
Article Type:	Protocol
Date Submitted by the Author:	18-Jan-2017
Complete List of Authors:	Casanova, Ludovic; INSERM, , , Marseille, France, UMR912 "Economics and Social Sciences Applied to Health & Analysis of Medical Information" (SESSTIM)<br>Cortaredona, Sebastien; INSERM, UMR912 "Economics and Social Sciences Applied to Health & Analysis of Medical Information" (SESSTIM), ; ORS PACA, Southeastern Health Regional Observatory,<br>Gaudart, Jean; SESSTIM, INSERM, UMR912 "Economics and Social Sciences Applied to Health & Analysis of Medical Information" (SESSTIM) Marseille, FR; Institut de recherche pour le developpement<br>Launay, Odile; Inserm, F-CRIN, Innovative clinical research network in vaccinology (I-REIVAC), Paris, France; Paris Descartes University, Sorbonne Paris Cité, Assistance Publique Hôpitaux de Paris, Cochin<br>Vanhems, Philippe; Université de Lyon; Université Lyon 1; CNRS UMR 5558, Biométrie et Biologie Evolutive, Epidémiologie et Santé Publique, Lyon, France, Hospices Civils de Lyon, Hôpital Edouard Herriot, Service d'Hygiène, Epidémiologie et Prévention, Lyon, France<br>Villani, Patrick; SESSTIM, INSERM, UMR912 "Economics and Social Sciences Applied to Health & Analysis of Medical Information" (SESSTIM), F-13385, Marseille, France; Observatoire Regional de la Sante Provence-Alpes-Cote d'Azur<br>Verger, Pierre; Aix Marseille Université , SESSTIM UMR 912, Inserm IRD; ORS PACA, Observatoire Régional de la Santé Provence-Alpes-Côte d'Azur
**Primary Subject Heading**:	Infectious diseases
Secondary Subject Heading:	Diabetes and endocrinology, Public health, Research methods, Evidence based practice
Keywords:	Public health < INFECTIOUS DISEASES, Epidemiology < INFECTIOUS DISEASES, General diabetes < DIABETES & ENDOCRINOLOGY

SCHOLARONE™
Manuscripts

TITLE PAGE

Title:

Effectiveness of seasonal influenza vaccination in patients with diabetes: protocol for a nested case-control study

Authors :

Ludovic Casanova[1,2,3,4], Sébastien Cortaredona[1,2,4], Jean Gaudart[1,4], Odile Launay[5,6], Philippe Vanhems[5,7], Patrick Villani[1,2,4], Pierre Verger[1,2,4,5]

Affiliations:

1 INSERM, UMR912 "Economics and Social Sciences Applied to Health & Analysis of Medical Information" (SESSTIM), F-13385, Marseille, France

2 ORS PACA, Southeastern Health Regional Observatory, 13006, Marseille, France

3 Aix Marseille University, Department of general practice, Marseille, France

4 Aix Marseille University, UMR_S912 (SESSTIM), IRD, F-13385, Marseille, France

5 Inserm, F-CRIN, Innovative clinical research network in vaccinology (I-REIVAC), Paris, France

6 Paris Descartes University, Sorbonne Paris Cité, Assistance Publique Hôpitaux de Paris, Cochin

7 Infection Control and Epidemiology Unit, Edouard Herriot Hospital, Hospices Civils de Lyon, Lyon, France

Corresponding author:

Ludovic Casanova, Observatoire régional de la santé Provence-Alpes-Côte d'Azur, 23 rue Stanislas Torrents, 13006 Marseille, France (Phone: +33 4 91 59 89 14, Fax: +33 4 91 59 89 18)

Email: ludovic.casanova@inserm.fr

The dates of the study : data necessary for the study will be available in June 2017.

Manuscrit:

3869 words (excluding references, tables, and abstract) / 3 tables

For peer review only

ABSTRACT

Introduction: Seasonal influenza vaccination (SIV) is recommended for people with diabetes, but its effectiveness has not been demonstrated. All of the available studies are observational and marred with the *healthy vaccinee bias*, that is, bias resulting from the generally better health behaviours practised by people who choose to be vaccinated against influenza, compared with those who do not. This protocol is intended to study the effectiveness of SIV in people with treated diabetes and simultaneously to control for bias.

Methods and analyses: This case-control study is nested in a historical cohort and is designed to study vaccine effectiveness (VE) assessed by morbidity, mortality, and anti-infective drug use. The cohort will comprise a representative sample of health insurance beneficiaries in France and will cover 10 consecutive epidemic seasons. It will include all patients reimbursed three separate times for drugs to treat diabetes. The first study of VE will use reasons for hospitalisation as the primary endpoint, and the second with the use of neuraminidase inhibitors and of antibiotics as the endpoints. A case will be defined as any person in the cohort reaching any endpoint at a given date. The case patient will be matched with the largest possible number of controls (individuals not reaching the endpoint by this date) according to the propensity score method with an optimal caliper width. A conditional logistic model will be used to estimate odds ratios to take into account both the matching and the repetition of measurements. The model will be applied separately during and outside of epidemic periods to estimate the residual confounding.

Ethics and dissemination: The study has been approved by the French Commission on Individual Data Protection and Public Liberties (ref: AT/CPZ/SVT/JB/DP/CR05222O). The study's findings will be published in peer-reviewed journals and disseminated at international conferences and through social media.

Abbreviations:

vaccine effectiveness (VE)

seasonal influenza vaccination (SIV)

general beneficiary sample (EGB)

10^{e} International Classification of Diseases (ICD-10)

relative risk (RR)

neuraminidase inhibitors (NAIs)

anatomical, treatment and chemical (ATC) classification

Keywords :

influenza vaccines

diabetes mellitus

nested case-control

propensity score ; protocol

For peer review only

Strengths and limitations of this study

- This study will generate new evidence to strengthen public policy recommendations for vaccination of diabetics, especially among adults of working-age.
- This is the first rigorous assessment of the effectiveness of influenza vaccination using the French National Health Insurance System database permitting analysis of about 40 000 patients over a ten year period
- This study explicitly accounts for healthy vaccinee bias that arises in observational studies where those patients who are more likely to be vaccinated are also more likely to prioritise other areas of their health. This bias may result in overestimation of vaccine effectiveness. In our study, we hypothesise that matching cases and controls by their propensity for other preventive behaviours may reduce this bias.
- The analysis includes a comparison of a non-epidemic control period to assess the presence of residual confounding associated with this healthy vaccinee bias.
- As pneumococcal pneumonia is a frequent complication of influenza, and this vaccine is recommended for most diabetics at risk of influenza, this study also considers the pneumococcal vaccination status for the five years before an event.
- By contrast to earlier studies, this study will use a more specific outcome measure for influenza.

For peer review only

INTRODUCTION

Seasonal influenza is an important cause of morbidity and mortality.[1] Its incidence is higher in children and young adults, but the morbidity and mortality it causes increase with age and in the presence of some chronic diseases, including diabetes.[2,3] The World Health Organization (WHO) recommends seasonal influenza vaccination (SIV) for patients with diabetes[4] because of their high risk of developing severe complications linked to this infection.[2,5] Most studies report that patients with diabetes and healthy patients have equivalent humoral responses to SIV.[6–8] Nonetheless, because influenza antibody titres do not correlate perfectly with clinical protection,[9] vaccine effectiveness (VE) studies are necessary. Recently two systematic reviews observed a lack of evidence of SIV effectiveness in patients with diabetes and concluded that observational studies are urgently needed to improve the methodological flaws of earlier studies.[10,11]

The measurement of SIV effectiveness in at-risk populations is subject to several methodological difficulties. Randomised clinical trials are not used to measure the effectiveness of recommended vaccines in the general population for ethical reasons; they have long been used and their recommendation indicates their effectiveness.[12] Seven observational studies[13–19] have been performed specifically in patients with diabetes to measure this effectiveness, two[13,15] in patients younger than 65 years. For reasons of feasibility and cost, none of these studies diagnosed influenza directly, by either PCR or serology. Instead they used indirect outcome measures, such as all-cause hospitalisation or all-cause death: this type of unspecific endpoint provides greater statistical power but also increases classification biases. Moreover, five of these studies did not take history of pneumococcal vaccination into account although influenza and pneumococcal pulmonary infection can present very similar clinical pictures, and vaccinations against these two diseases have common determinants of adherence.[19,20] Finally, all seven studies are subject to the *healthy vaccinee bias:*[21,22] this bias results from the fact that people who are vaccinated against influenza are, on the whole, more attentive to their health, visit their physicians more often, and probably have better controlled diabetes than unvaccinated individuals. These confounding factors may explain the lower levels of morbidity and mortality observed in vaccinated individuals, independently of the vaccine's protective effect. Accordingly, vaccinated patients have lower rates of morbidity and mortality during non-epidemic periods (that is, seasons during which the influenza virus is not circulating; several authors describe these lower rates as residual confounding.[10,11]

This article presents the protocol of an observational study which has as its principal objective the assessment of SIV effectiveness in patients with treated type 1 and type 2 diabetes while attempting to minimize the preceding methodological limitations. The protocol conducts two nested case-control studies within the very large cohort of beneficiaries of the principal health insurance fund in France. The first study will analyse VE by the reduction in hospitalisations for clinical pictures suggestive of influenza (composite criterion) and for all-cause hospitalisation, and the second by the reduction in outpatient use of neuraminidase-inhibiting antiviral agents (NAIs) and of the antibiotics indicated for treatment of post-influenza lung diseases.

For peer review only

METHODS AND ANALYSES

Database

The study will be performed by analysing reimbursement data from the French national health insurance fund. Data will be anonymously extracted from the fund's permanent sample of beneficiaries (“Echantillon Généraliste des Bénéficiaires”, EGB) database. The EGB, created in 2003, is a permanent representative sample of persons affiliated to the French health insurance system, a cohort of beneficiaries for whom information on health-care utilisation is conserved for 20 years. These data can thus be used to assess longitudinal utilisation patterns for research purposes. It is obtained by a 1/97th national random sampling with stratification for age and sex as described in more detail previously.[23] The identification of persons included in the sample is protected by a two-level cryptographic anonymisation process. The EGB is obtained from the reimbursement databases of the major public health insurance funds (salaried workers), which record information on the health-care utilisation of nearly 85% of the general population in France. On 1 January, 2016, the EGB database included 675 906 individuals for whom it contains the following information: age, sex, all reimbursements for medical expenses for outpatient care, and their “chronic disease status“ with its diagnostic codes according to the 10th revision of the International Classification of Diseases (ICD-10). “Chronic disease status” is attributed to people with specific and expensive chronic diseases defined by the French health insurance system and makes them eligible for 100% reimbursement of the costs of their treatment as described in more detail previously.[24] Since January 2006, the EGB has also provided access to all episodes of hospitalisation in public and private hospitals and to their associated discharge diagnoses, coded according to ICD-10.

Definition of the study cohort

This historical cohort of people treated for diabetes will be followed from 12 October, 2006 (date that the 2006-2007 campaign vaccination started), through 9 October, 2016 (day before the start of the 2016-2017 campaign vaccination). Patients will be included only if they were in the database for at least 3 years before their follow-up begins. People treated for diabetes will be identified according to the following criteria, previously used in epidemiologic studies: they are listed in the databases as having received at least three deliveries of oral diabetes drugs or insulin on three distinct dates in the 12 months before inclusion.[25] For patients to whom oral diabetes drugs were dispensed in the year before the start of follow-up (prevalent diabetes at inclusion), the inclusion date will be the study start date. For new cases of treated diabetes (incident cases of treated diabetes during the study period), the date of inclusion will be the date of the first delivery of diabetes drugs. Patients who change insurance funds or die during follow-up will be censored.

Years of follow-up

For peer review only

The maximum duration of follow-up will be 10 years. A year of follow-up will begin on the first day of the SIV campaign of the year *n* and will end the day preceding the start of the campaign for the year *n*+1. The French Ministry of Defence sets the dates that SIV campaigns begin each year (table 1). The covariables will be assessed at the beginning of each year of follow-up (baseline variables) and considered stable through the end of that year.

SIV status

During each year of follow-up, SIV status will be defined as positive 8 days after the day the vaccine was dispensed to the patient at the pharmacy (the minimum interval before protective antibodies appear[26–28]). This delivery will be identified by its code in the Anatomical, Treatment and Chemical classification for influenza vaccines (ATC Code J07BBxx). We hypothesise that patients will be vaccinated the day they purchase the vaccine at the pharmacy. A sensitivity analysis will test different intervals between the vaccine purchase date and the vaccination date to verify the impact of this hypothesis on the results.

Cohort-nested case-control study

A case will be defined as any person in the cohort presenting any endpoint at a given date. For each case, the controls will be defined as all patients not presenting that endpoint up to the date it occurred for the case. A patient can be chosen as a control numerous times. Any patient who presents an endpoint during a given year becomes a case and is thus excluded from the control pool until the beginning of the next year. Controls will be matched to cases according to the propensity score method.[29] The propensity score is a one-dimensional summary of all of the matching variables and will furnish an estimate of the probability of being vaccinated as a function of several specific variables. It will be calculated by logistic regression with vaccination as the dependent variable. The model's explanatory variables will be: the date of cohort entry, to control for variations over time (+/- 6 months), age, sex, adherence to secondary prevention examinations for diabetes follow-up, visits to general practitioners, history of pneumococcal vaccination, comorbidities, diabetes severity, hospitalisation in the previous year, and SIV history. In the most common implementation of propensity-score matching, pairs of cases and controls are formed when their propensity scores differ by a pre-specified maximum amount. To minimise means or risk differences, we will match for the logit of the propensity score, using calipers of width equal to 0.2 of the standard deviation of the logit of the propensity score [30]. The adjusted odds ratio from this conditional logistic regression will be corrected to approach a relative risk (RR) according to the method of Zhang and Yu [31]. The following formula will be used to calculate the VE:

$$VE\ (\%) = (1 - RR) * 100$$

First study: endpoints based on hospitalisation and its causes

The principal endpoint will be hospitalisation for a clinical picture suggestive of influenza or an indirect complication (table 2). Specifically, it will be the composite criterion of the ICD-10 codes used by Lau

et al. in 2013 to study VE.[15] It is based on the reasons for hospitalisation of patients referred by private-practice physicians to hospital emergency departments for influenza. The date of the onset of the criterion will be the first day of hospitalisation. The secondary endpoint will be death or hospitalisation from all causes combined, excluding planned admissions.

Second study: Endpoints based on outpatient prescriptions

The principal endpoint will be the dispensing by a private pharmacy of an NAI (ATC Code: J05AH, oseltamivir or zanamivir). NAIs are systemic antiviral treatments used to cure seasonal influenza in the 48 hours after its first symptoms in individuals at risk of complications, including patients with diabetes, but is prescribed on an outpatient basis to only approximately 25% of patients for whom it is indicated.[32] This endpoint makes it possible to identify a portion of the influenza cases receiving outpatient care.

The secondary endpoint will be the delivery in a private pharmacy of one of the antibiotic treatment recommended [33] as a first- or second-line treatment for cases of acute, community-acquired post-influenza lung diseases: amoxicillin, amoxicillin/clavulanic acid (ATC Code: J01CR, excluding the following compounds with the same code: ticarcillin/clavulanic acid, ampicillin/sulbactam and piperacillin/tazobactam), ceftriaxone (ATC Code: J01DD04), pristinamycin (ATC Code: J01FG01), levofloxacin (ATC Code: S01AE05), and telithromycin (ATC Code: J01FA15). These recommendations did not change during the study period.

Statistical analyses

To take into account the variable number of controls per case, the descriptive statistics will be weighted by the inverse of the number of controls in those analyses. As recommended for the nested case-control study design,[34] a conditional logistic model will be used to calculate the odds ratio to take into account both the matching of individuals in the analysis and the repetition of measurements. Recommendations for use of propensity score matching suggest adjusting for the propensity score, because matching plus adjustment reduces the previous imbalance between two cases and controls more effectively than matching alone.[35] Adjusting for the propensity score thus improves the estimate of the intervention effect.

This model will be applied separately during epidemic periods and non-epidemic periods to estimate possible residual confounding due to the healthy vaccinee bias. Each year, the epidemic period will be defined as the period during which the incidence of influenza-like illnesses (ILIs) exceeds the epidemic thresholds defined from influenza surveillance data in France (table 1).[36–38] The non-epidemic periods will be those during which there is no recorded circulation of the virus: 15 May to 31 July each year since 1985 (websenti.u707.jussieu.fr).

Moreover, the analyses will be stratified for age: they will be performed separately for patients aged 65 years or older and for those younger than 65 years. SIV is doubly indicated for patients with diabetes aged 65 years or older — for both their age and their chronic condition. Vaccination coverage of people with diabetes younger than 65 years is lower than that of older patients,[20] and clear VE data

For peer review only

lack for this younger age group, who account for nearly half the patients with diabetes treated pharmacologically in France.[39]

Justification of the variables included in the propensity score calculation

1/ Age

The risks of hospitalisation and influenza-related complications increase with age, while influenza incidence decreases with age. Age will be defined in the following classes: [18-35[; [35-45[; [45-55[; [55-65[; [65-75[; [75-85[; >85. Including age in the calculation of the propensity score enables consideration of the immunosenescence that reduces VE in older subjects, through its impairment of response to new antigens and its reduction in immune memory.[40,41]

2/ Sex

At the same age, men with diabetes are at greater risk of hospitalisation than women. Several studies report an excess risk of influenza-related complications among men, although the evidence is not definitive. Men appear to have better SIV coverage[42] and are vaccinated with greater regularity.[20]

3/ Adherence to examinations for secondary prevention in diabetes follow-up

Adherence to SIV depends, in part, on the behaviour and attitudes of both the patient and the general practitioner in the follow-up of chronic diseases.[43] Patients with diabetes who are vaccinated against influenza are also more adherent in the follow-up of their diabetes than those not vaccinated;[44], this can affect both the control and complications of their diabetes. In people with diabetes, inadequate disease control is associated with 80% of all-cause hospitalisation.[13,16] These points partly explain the healthy vaccinee bias when the VE is calculated from hospital admissions. A proxy variable for adherence with secondary prevention of people with diabetes will be constructed according to a previously published method.[45] This adherence score ranges from 0 to 5 and attributes a value of 1 for each of these examinations performed during the past three years: HbA1C, microalbuminuria, LDL-cholesterol, ocular fundus, podiatrist consultation. This score has been shown to be partially correlated with the frequency of general practitioner visits, but not collinear with it.[46]

4/ Frequency of visits to a general practitioner in the past year

The probability of vaccination against influenza increases with the number of visits to general practitioners.[20,47] For patients 65 years or older with diabetes, Rodriguez et al[19] found a mean of 14 annual GP consultations among the unvaccinated patients versus 20 among those vaccinated. GP consultations can modify patients' health care pathway by reducing the risk of hospitalisation, for example, by earlier care for influenza (treatment by NAIs in the first 48 first hours of an ILI[32]). At the same time, it enables the best possible diabetes control through more regular monitoring. Preceding studies of VE show that it is preferable to include it as a continuous variable.[13,15]

For peer review only

5/ History of pneumococcal vaccination

Patients with diabetes are at increased risk of invasive pneumococcal infections.[48–50] Adherence to SIV and to pneumococcal vaccination have common determinants,[47] with pneumococcal vaccination coverage is best in patients vaccinated against influenza.[19] Without adjustment for history of this vaccination, SIV effectiveness may be overestimated. The official recommendation of routine pneumococcal vaccination in patients with diabetes dates back only to 2009 in France but such vaccination took place before then. To construct this adjustment variable, we will consider as vaccinated all patients with at least one delivery of the pneumococcal vaccine (*ATC Code*: J07A L01) in the 5 years before the year under consideration (period of persistence of the protective antibody level[51,52]). Pneumococcal vaccination status will be considered positive the day the vaccine is dispensed at the pharmacy. Like influenza vaccination status, pneumococcal vaccination status will not be a baseline value but may vary over a year of follow-up. Because of this variable's importance for potential confounding, an interaction term will be added to the models. Should the latter be significant, an analysis stratified by pneumococcal vaccination will be performed to estimate SIV effectiveness in patients with pneumococcal vaccination within the past 5 years and in those vaccinated either never or not in the past 5 years.

6/ Diabetes severity

The severity of diabetes is simultaneously positively associated with the risk of hospitalisation for infectious causes,[53] including seasonal influenza,[2,54] and with vaccination coverage.[20] It is therefore a confounding factor. Because the EGB databases do not include any individual clinical or laboratory variable to measure diabetes severity directly, it will be approached indirectly by two different variables:

- The intensity of the treatments prescribed: a single oral diabetes medication for mild diabetes, two different such medications for moderate diabetes, and more than two different drugs and/or insulin for severe diabetes;
- Time since diagnosis of diabetes is associated with the risks of influenza complications and of immunodeficiency secondary to chronic hyperglycaemia. A proxy variable for this duration will be the estimated as of 2003 (date of earliest EGB data).

7/ Comorbidities

Patients with diabetes and at least one comorbidity have both better vaccination coverage[20] and a higher risk of influenza complications: if this "confounding by indication" is not taken into account, it can result in underestimating SIV effectiveness.[22] For each patient, we have diagnoses (coded with ICD-10) associated with potential chronic disease status, in addition to diabetes. To use this variable most productively, we plan to regroup them into four major disease groups (table 3): respiratory diseases, cardiovascular diseases, kidney diseases, and neoplasms and/or immunodeficiencies.

8/ Hospitalisation in the preceding year

For peer review only

Adjustment for this variable is also intended to improve control for confounding by indication. Hospitalisation during the preceding year can result in behavioural changes in subsequent years.[55] We can expect an excess of hospitalisations in vaccinated patients, associated not with influenza complications but rather with fragility related to health status. This variable will be constructed dichotomously and separately for each year of follow-up: no hospitalisation or at least one hospitalisation for any cause (excluding planned hospitalisations) during the year preceding the year of follow-up considered.

9/ History of SIV

Currently, the literature about the effect of repeated SIV is contradictory. A meta-analysis published in 1999 suggested that repeated SIV does not affect its effectiveness.[56] A recent study of vaccinated patients showed a reduction in influenza-positive respiratory infections that was greatest among those not vaccinated for the preceding 5 years.[57] Nonetheless other work has shown that the serologic protection against influenza caused by vaccination may persist from one year to another if the virus does not mutate,[53] as it regularly does in practice. Accordingly, one study observed that mortality fell by 15% more in the group regularly vaccinated against influenza than in the newly vaccinated group.[59] We have therefore chosen to adjust for the presence of SIV during the two years preceding the year under consideration, as earlier studies have done.[13]

Discussion

Strengths and limitations of the study design

The design of a case-control study nested in a cohort is a sampling technique based on the principle of sampling within the group at risk ("risk set sampling") and simplifies consideration of exposures and covariables that vary over time. This design optimises the use of the available data by making it possible for a patient to be, at different times, a control and a case and thus facilitates the analysis of infrequent events. The hospitalisation rate for influenza in patients with a chronic condition is around 2% in France.[60] This design also enables the study of rare endpoints. It should also allow separate analyses of data restricted to the epidemic period and to the non-epidemic season despite the resulting reduction in the number of observations. Another useful point of this design is the improved comparability between cases and controls made possible by propensity score matching, a method producing the lowest mean quadratic error when large numbers of controls are available.[29,61] Propensity score matching allows the comparison of patients with the same probability of being vaccinated and thus reduces the healthy vaccinee bias.

Strengths and limitations of endpoints related to hospitalisation and its causes

Hospitalisation for typical clinical influenza (i.e., ILI?) or its complications will be the endpoint most specific for influenza; this criterion is relatively independent of the healthy vaccinee bias and helps to minimise residual confounding. It is therefore probable that we will not observe differences in this endpoint during the non-epidemic season. Inversely the endpoints of death and all-cause hospitalisation are more sensitive to this bias. Among patients with diabetes aged 65 years or older,

SIV also appears to reduce all-cause mortality by 38% or more.[10] Nonetheless the excess mortality associated with influenza is estimated at 5 to 10%.[62,63] A substantial portion of the reduction above is therefore linked to unobserved factors. Most studies applying this criterion have found that VE persists during the off-season. This endpoint has been used as a marker of residual confounding; its failure to appear, contrary to earlier studies, will enable us to shed light on how this design affects it.

Strengths and limitations of the endpoints based on outpatient medication use

In patients with diabetes younger than 65 years, the low rate of hospitalisation for ILI makes it difficult to measure VE. The two studies that studied VE with this criterion in this population found no significant effect.[13,15] No study has yet used outpatient NAI use to measure VE, but evidence indicates its relevance. NAIs are a specific treatment for the influenza virus. Models of chronological series of influenza activity in different countries report strong correlations between the official surveillance of sentinel physicians and pharmacy NAI sales,[64–67] especially for patients of working age. The disadvantages of this endpoint are associated with the multiple other therapeutic indications for NAIs, including: i) post-exposure prophylaxis of seasonal influenza in adult after contact with a clinically diagnosed case in the family and in other exceptional situations; ii) prophylaxis, for both pandemic situations and seasonal influenza during epidemic periods when the circulating viral strains do not match the vaccine. No data describe the proportion of NAIs dispensed for these two indications. The other limitation is related to the lack of specificity of the initial clinical presentation of influenza. Work has shown that primary-care physicians' NAI prescriptions are suboptimal,[68] but no evidence shows that this bias is differential.

Antibiotic prescriptions for influenza complications are the second principal component of outpatient care for influenza accessible in the database. The principal complication of influenza in patients with diabetes is a pneumococcal lung infection. Numerous studies have shown that seasonal influenza epidemics are strongly associated with increased use of antibiotics, especially aminopenicillins and macrolides.[69] Thus, influenza vaccination should result in a reduction in antibiotic use, which has already been used as an indirect endpoint for assessing SIV effectiveness: in Ontario (Canada), antibiotic prescriptions fell after the introduction of universal free vaccination against influenza.[70,71]

Strengths and limitations associated with the database

This will be the first study of the effectiveness of SIV performed in France from a nationwide healthcare-related administrative database. We know that the intensity of epidemics varies from year to year due to the circulating serotypes, the population's history of exposure, and variations in SIV effectiveness, due in turn to antigenic modifications of influenza virus strains that can result in a poor match between the circulating strains and those contained in the vaccine. The impact of these mismatches varies highly according to the number and extent of the mutations and to whether or not they affect the circulating strain predominantly. Accordingly, mismatches for the A(H3N2) virus during the 2012-2013 and 2014-2015 seasons explain the poor vaccine effectiveness observed among the elderly.[72] The use of data from 10 epidemic seasons will allow us to smooth this variability;

For peer review only

moreover, this study will provide the largest number of seasons of observation of patients with diabetes than any previously published study.

For peer review only

FOOTNOTES

Acknowledgments : None

Contributors: L. CASANOVA (MD PhD student) drafted this study protocol under the direction of P. VERGER (MD PhD) and P. VILLANI (MD PhD). S. CORTAREDONA (MD statistician) contributed to the data management and construction of the statistical model. J. GAUDART (MD PhD) provided his expertise for the design of the nested case-control study. P. VANHEMS (MD PhD) and Odile LAUNAY (MD PhD) provided their expertise about vaccination and vaccine effectiveness. All the authors participated in the drafting and revision of the manuscript. All authors were major contributors in interpretation of data and in writing the manuscript. All authors approved the final version and agree to be accountable for the contents and integrity of this manuscript.

Competing interests: None.

Funding: This research received no specific grant from any funding agency in the public, commercial or not-for-profit sectors

Ethics approval: Article 3 of the decree dated 20 June 2005 concerning the implementation of the interregional health insurance information system (updated by the decree dated 11 July 2012) foresaw the establishment of a sample of patients affiliated with the French national health insurance system, to be called the general beneficiary sample (EGB). Data for this sample may be conserved for 20 years after the day's date. This panel is administered by the CNAM-TS and has been approved by the CNIL (CNIL (French Commission on Individual Data Protection and Public Liberties, study registered under number AT/CPZ/SVT/JB/DP/CR05222O of 14 June 2005 and number DP/CR071761 of 28 August 2007. French law does not require authorisation of an ethics committee for the use of routinely collected data by authorised research teams (Official Journal of the Republic of French, 2012). The mixed research unit (AMU/Inserm/IRD) that will supervise this study is so authorised.

For peer review only

REFERENCES

1 Dawood FS, Iuliano AD, Reed C, *et al.* Estimated global mortality associated with the first 12 months of 2009 pandemic influenza A H1N1 virus circulation: a modelling study. *Lancet Infect Dis* 2012;**12**:687–95. doi:10.1016/S1473-3099(12)70121-4

2 Muller LMAJ, Gorter KJ, Hak E, *et al.* Increased risk of common infections in patients with type 1 and type 2 diabetes mellitus. *Clin Infect Dis* 2005;**41**:281–8. doi:10.1086/431587

3 Isotani H. Use of influenza vaccine in patients with diabetes, particularly those with complications of cardiac or renal disease. *Intern Med* 2000;**39**:862.

4 World Health Organization. Fact sheet on influenza. 2014. WHO. http://www.who.int/mediacentre/factsheets/fs211/en/ (accessed 21 Jul2015).

5 Mertz D, Kim TH, Johnstone J, *et al.* Populations at risk for severe or complicated influenza illness: systematic review and meta-analysis. *BMJ* 2013;**347**:f5061.

6 Frasca D, Diaz A, Romero M, *et al.* Young and elderly patients with type 2 diabetes have optimal B cell responses to the seasonal influenza vaccine. *Vaccine* 2013;**31**:3603–10. doi:10.1016/j.vaccine.2013.05.003

7 Pozzilli P, Gale EA, Visalli N, *et al.* The immune response to influenza vaccination in diabetic patients. *Diabetologia* 1986;**29**:850–4.

8 el-Madhun AS, Cox RJ, Seime A, *et al.* Systemic and local immune responses after parenteral influenza vaccination in juvenile diabetic patients and healthy controls: results from a pilot study. *Vaccine* 1998;**16**:156–60.

9 Reber A, Katz J. Immunological assessment of influenza vaccines and immune correlates of protection. *Expert Rev Vaccines* 2013;**12**:519–36. doi:10.1586/erv.13.35

10 Remschmidt C, Wichmann O, Harder T. Vaccines for the prevention of seasonal influenza in patients with diabetes: systematic review and meta-analysis. *BMC Med* 2015;**13**:53. doi:10.1186/s12916-015-0295-6

11 Casanova L, Gobin N, Villani P, *et al.* Bias in the measure of the effectiveness of seasonal influenza vaccination among diabetics. *Prim Care Diabetes* Published Online First: 8 June 2016. doi:10.1016/j.pcd.2016.05.005

12 Fiore AE, Uyeki TM, Broder K, *et al.* Prevention and control of influenza with vaccines: recommendations of the Advisory Committee on Immunization Practices (ACIP), 2010. *MMWR Recomm Rep* 2010;**59**:1–62.

13 Akker IL-V den, Verheij TJM, Buskens E, *et al.* Clinical Effectiveness of First and Repeat Influenza Vaccination in Adult and Elderly Diabetic Patients. *Dia Care* 2006;**29**:1771–6. doi:10.2337/dc05-2517

14 Wang I-K, Lin C-L, Chang Y-C, *et al.* Effectiveness of influenza vaccination in elderly diabetic patients: a retrospective cohort study. *Vaccine* 2013;**31**:718–24. doi:10.1016/j.vaccine.2012.11.017

15 Lau D, Eurich DT, Majumdar SR, *et al.* Effectiveness of influenza vaccination in working-age adults with diabetes: a population-based cohort study. *Thorax* 2013;**68**:658–63. doi:10.1136/thoraxjnl-2012-203109

16 Colquhoun AJ, Nicholson KG, Botha JL, *et al.* Effectiveness of influenza vaccine in reducing hospital admissions in people with diabetes. *Epidemiol Infect* 1997;**119**:335–41.

For peer review only

17 Heymann AD, Shapiro Y, Chodick G, *et al.* Reduced Hospitalizations and Death Associated With Influenza Vaccination Among Patients With and Without Diabetes. *Dia Care* 2004;**27**:2581–4. doi:10.2337/diacare.27.11.2581

18 Hak E, Nordin J, Wei F, *et al.* Influence of high-risk medical conditions on the effectiveness of influenza vaccination among elderly members of 3 large managed-care organizations. *Clin Infect Dis* 2002;**35**:370–7. doi:10.1086/341403

19 Rodriguez-Blanco T, Vila-Corcoles A, de Diego C, *et al.* Relationship between annual influenza vaccination and winter mortality in diabetic people over 65 years. *Hum Vaccin Immunother* 2012;**8**:363–70. doi:10.4161/hv.18548

20 Verger P, Cortaredona S, Pulcini C, *et al.* Characteristics of patients and physicians correlated with regular influenza vaccination in patients treated for type 2 diabetes: a follow-up study from 2008 to 2011 in southeastern France. *Clinical Microbiology and Infection*;**0**. doi:10.1016/j.cmi.2015.06.017

21 Hak E, Verheij TJM, Grobbee DE, *et al.* Confounding by indication in non-experimental evaluation of vaccine effectiveness: the example of prevention of influenza complications. *J Epidemiol Community Health* 2002;**56**:951–5. doi:10.1136/jech.56.12.951

22 Remschmidt C, Wichmann O, Harder T. Frequency and impact of confounding by indication and healthy vaccinee bias in observational studies assessing influenza vaccine effectiveness: a systematic review. *BMC Infect Dis* 2015;**15**:429. doi:10.1186/s12879-015-1154-y

23 Tuppin P, de Roquefeuil L, Weill A, *et al.* French national health insurance information system and the permanent beneficiaries sample. *Rev Epidemiol Sante Publique* 2010;**58**:286–90. doi:10.1016/j.respe.2010.04.005

24 Cortaredona S, Pambrun E, Verdoux H, *et al.* Comparison of pharmacy-based and diagnosis-based comorbidity measures from medical administrative data. *Pharmacoepidemiol Drug Saf* Published Online First: 2 December 2016. doi:10.1002/pds.4146

25 Tiv M, Viel J-F, Mauny F, *et al.* Medication adherence in type 2 diabetes: the ENTRED study 2007, a French Population-Based Study. *PLoS ONE* 2012;**7**:e32412. doi:10.1371/journal.pone.0032412

26 Halliley JL, Kyu S, Kobie JJ, *et al.* Peak frequencies of circulating human influenza-specific antibody secreting cells correlate with serum antibody response after immunization. *Vaccine* 2010;**28**:3582–7. doi:10.1016/j.vaccine.2010.02.088

27 Cox RJ, Brokstad KA, Zuckerman MA, *et al.* An early humoral immune response in peripheral blood following parenteral inactivated influenza vaccination. *Vaccine* 1994;**12**:993–9.

28 Sasaki S, He X-S, Holmes TH, *et al.* Influence of prior influenza vaccination on antibody and B-cell responses. *PLoS ONE* 2008;**3**:e2975. doi:10.1371/journal.pone.0002975

29 Garrido MM, Kelley AS, Paris J, *et al.* Methods for Constructing and Assessing Propensity Scores. *Health Serv Res* 2014;**49**:1701–20. doi:10.1111/1475-6773.12182

30 Austin PC. Optimal caliper widths for propensity-score matching when estimating differences in means and differences in proportions in observational studies. *Pharm Stat* 2011;**10**:150–61. doi:10.1002/pst.433

31 Zhang J, Yu KF. What's the relative risk? A method of correcting the odds ratio in cohort studies of common outcomes. *JAMA* 1998;**280**:1690–1.

32 Dobson J, Whitley RJ, Pocock S, *et al.* Oseltamivir treatment for influenza in adults: a meta-analysis of randomised controlled trials. *The Lancet* 2015;**385**:1729–37. doi:10.1016/S0140-6736(14)62449-1

For peer review only

33 AFSSAPS. Antibiotics in lower respiratory tract infections in adults and children. French Guidelines. October 2005.

34 Kim MY, Zeleniuch-Jacquotte A. Correcting for measurement error in the analysis of case-control data with repeated measurements of exposure. *Am J Epidemiol* 1997;**145**:1003–10.

35 Caliendo M, Kopeinig S. Some Practical Guidance for the Implementation of Propensity Score Matching. *Journal of Economic Surveys* 2008;**22**:31–72. doi:10.1111/j.1467-6419.2007.00527.x

36 Costagliola D, Flahault A, Galinec D, *et al.* A routine tool for detection and assessment of epidemics of influenza-like syndromes in France. *Am J Public Health* 1991;**81**:97–9.

37 Serfling RE. Methods for current statistical analysis of excess pneumonia-influenza deaths. *Public Health Rep* 1963;**78**:494–506.

38 Stroup DF, Williamson GD, Herndon JL, *et al.* Detection of aberrations in the occurrence of notifiable diseases surveillance data. *Stat Med* 1989;**8**:323-329-332.

39 Fosse-Edorh S, Fagot-Campagna A, Detournay B, *et al.* Impact of socio-economic position on health and quality of care in adults with Type 2 diabetes in France: the Entred 2007 study. *Diabet Med* Published Online First: 17 April 2015. doi:10.1111/dme.12783

40 Goronzy JJ, Weyand CM. Understanding immunosenescence to improve responses to vaccines. *Nat Immunol* 2013;**14**:428–36. doi:10.1038/ni.2588

41 Lang PO, Govind S, Bokum AT, *et al.* Immune senescence and vaccination in the elderly. *Curr Top Med Chem* 2013;**13**:2541–50.

42 Jimenez-Trujillo I, López-de Andrés A, Hernández-Barrera V, *et al.* Influenza vaccination coverage rates among diabetes sufferers, predictors of adherence and time trends from 2003 to 2010 in Spain. *Hum Vaccin Immunother* 2013;**9**:1326–32. doi:10.4161/hv.23926

43 van Essen GA, Kuyvenhoven MM, de Melker RA. Compliance with influenza vaccination. Its relation with epidemiologic and sociopsychological factors. *Arch Fam Med* 1997;**6**:157–162; discussion 163.

44 Achtymichuk KA, Johnson JA, Al Sayah F, *et al.* Characteristics and health behaviors of diabetic patients receiving influenza vaccination. *Vaccine* 2015;**33**:3549–55. doi:10.1016/j.vaccine.2015.05.047

45 Gregg EW, Karter AJ, Gerzoff RB, *et al.* Characteristics of insured patients with persistent gaps in diabetes care services: the Translating Research into Action for Diabetes (TRIAD) study. *Med Care* 2010;**48**:31–7. doi:10.1097/MLR.0b013e3181bd4783

46 Ricci P, Chantry M, Detournay B. Coûts des soins remboursés par l'Assurance maladie aux personnes traitées pour diabète : Études Entred 2001 et 2007. *BEH n°42-43* 2009;:464–8.

47 Clancy U, Moran I, Tuthill A. Prevalence and predictors of influenza and pneumococcal vaccine uptake in patients with diabetes. *Ir Med J* 2012;**105**:298–300.

48 Thomsen RW, Hundborg HH, Lervang H-H, *et al.* Risk of community-acquired pneumococcal bacteremia in patients with diabetes: a population-based case-control study. *Diabetes Care* 2004;**27**:1143–7.

49 Klemets P, Lyytikäinen O, Ruutu P, *et al.* Risk of invasive pneumococcal infections among working age adults with asthma. *Thorax* 2010;**65**:698–702. doi:10.1136/thx.2009.132670

50 Valdez R, Narayan KM, Geiss LS, *et al.* Impact of diabetes mellitus on mortality associated with pneumonia and influenza among non-Hispanic black and white US adults. *Am J Public Health* 1999;**89**:1715–21.

For peer review only

51 Musher DM, Manof SB, Liss C, *et al.* Safety and antibody response, including antibody persistence for 5 years, after primary vaccination or revaccination with pneumococcal polysaccharide vaccine in middle-aged and older adults. *J Infect Dis* 2010;**201**:516–24. doi:10.1086/649839

52 Fisman DN, Abrutyn E, Spaude KA, *et al.* Prior pneumococcal vaccination is associated with reduced death, complications, and length of stay among hospitalized adults with community-acquired pneumonia. *Clin Infect Dis* 2006;**42**:1093–101. doi:10.1086/501354

53 Bertoni AG, Saydah S, Brancati FL. Diabetes and the risk of infection-related mortality in the U.S. *Diabetes Care* 2001;**24**:1044–9.

54 Peleg AY, Weerarathna T, McCarthy JS, *et al.* Common infections in diabetes: pathogenesis, management and relationship to glycaemic control. *Diabetes Metab Res Rev* 2007;**23**:3–13. doi:10.1002/dmrr.682

55 Hottes TS, Skowronski DM, Hiebert B, *et al.* Influenza vaccine effectiveness in the elderly based on administrative databases: change in immunization habit as a marker for bias. *PLoS ONE* 2011;**6**:e22618. doi:10.1371/journal.pone.0022618

56 Beyer WE, de Bruijn IA, Palache AM, *et al.* Protection against influenza after annually repeated vaccination: a meta-analysis of serologic and field studies. *Arch Intern Med* 1999;**159**:182–8.

57 McLean HQ, Thompson MG, Sundaram ME, *et al.* Impact of repeated vaccination on vaccine effectiveness against influenza A(H3N2) and B during 8 seasons. *Clin Infect Dis* 2014;**59**:1375–85. doi:10.1093/cid/ciu680

58 Petrie JG, Ohmit SE, Johnson E, *et al.* Persistence of Antibodies to Influenza Hemagglutinin and Neuraminidase Following One or Two Years of Influenza Vaccination. *J Infect Dis* 2015;**212**:1914–22. doi:10.1093/infdis/jiv313

59 Voordouw ACG, Sturkenboom MCJM, Dieleman JP, *et al.* Annual Revaccination Against Influenza and Mortality Risk in Community-Dwelling Elderly Persons. *JAMA November 3, 2004* 2004;**292**:2089–95.

60 Groupe de Travail. [Taking care of influenza in the context of a pandemic in 2005. Full text]. *Med Mal Infect* 2005;**35 Suppl 4**:S245-273.

61 Kwiatkowski F, Slim K, Verrelle P, *et al.* [Propensity score: interest and limits]. *Bull Cancer* 2007;**94**:680–6.

62 Horcajada JP, Pumarola T, Martínez JA, *et al.* A nosocomial outbreak of influenza during a period without influenza epidemic activity. *Eur Respir J* 2003;**21**:303–7.

63 Berg HF, Van Gendt J, Rimmelzwaan GF, *et al.* Nosocomial influenza infection among post-influenza-vaccinated patients with severe pulmonary diseases. *J Infect* 2003;**46**:129–32.

64 Centers for Disease Control and Prevention (CDC). Value of pharmacy-based influenza surveillance - Ontario, Canada, 2009. *MMWR Morb Mortal Wkly Rep* 2013;**62**:401–4.

65 Yoshida M, Matsui T, Ohkusa Y, *et al.* Seasonal influenza surveillance using prescription data for anti-influenza medications. *Jpn J Infect Dis* 2009;**62**:233–5.

66 Sugawara T, Ohkusa Y, Ibuka Y, *et al.* Real-time prescription surveillance and its application to monitoring seasonal influenza activity in Japan. *J Med Internet Res* 2012;**14**:e14. doi:10.2196/jmir.1881

67 Liu C-H, Wang J-L, Su C-P, *et al.* Oseltamivir use and outcomes during the 2009 influenza A H1N1 pandemic in Taiwan. *BMC Public Health* 2013;**13**:646. doi:10.1186/1471-2458-13-646

For peer review only

68 Havers F, Thaker S, Clippard JR, *et al.* Use of influenza antiviral agents by ambulatory care clinicians during the 2012-2013 influenza season. *Clin Infect Dis* 2014;**59**:774–82. doi:10.1093/cid/ciu422

69 Sun L, Klein EY, Laxminarayan R. Seasonality and Temporal Correlation between Community Antibiotic Use and Resistance in the United States. *Clin Infect Dis* 2012;**55**:687–94. doi:10.1093/cid/cis509

70 Kwong JC, Maaten S, Upshur REG, *et al.* The Effect of Universal Influenza Immunization on Antibiotic Prescriptions: An Ecological Study. *Clin Infect Dis* 2009;**49**:750–6. doi:10.1086/605087

71 Glezen WP. Benefits of a Universal Influenza Immunization Program: More than the Reduction in the Use of Antibiotics. *Clin Infect Dis* 2009;**49**:757–8. doi:10.1086/605088

72 Centers for Disease Control and Prevention (CDC). Interim adjusted estimates of seasonal influenza vaccine effectiveness - United States, February 2013. *MMWR Morb Mortal Wkly Rep* 2013;**62**:119–23.

For peer review only

Table 1. Description of the 10 study years: date the vaccination campaign began, characteristics of the epidemic and control periods.

	Year	Campaign start date*	Epidemic period					Control period	
			Start	End	Duration	Peak**	Serotypes***	Start	End
1	2006/2007	12/10/06	2007w03	2007w09	7	2007w06	AH3N2	2007w18	2007w30
2	2007/2008	28/09/07	2008w02	2008w10	9	2008w06	AH1N1/B	2008w18	2008w30
3	2008/2009	10/10/08	2008w51	2009w08	10	2009w04	AH3N2	2009w18	2009w30
4	2009/2010	01/09/09	2009w37	2009w52	16	2009w49	AH1N1	2010w18	2010w30
5	2010/2011	24/09/10	2010w51	2011w07	9	2011w01	AH1N1	2011w18	2011w30
6	2011/2012	29/09/11	2012w05	2012w12	8	2012w08	AH3N2	2012w18	2012w30
7	2012/2013	28/09/12	2012w51	2013w11	13	2013w05	AH1N1/B	2013w18	2013w30
8	2013/2014	11/10/13	2014w05	2014w10	6	2014w07	AH1N1/AH3N2	2014w18	2014w30
9	2014/2015	16/10/14	2015s03	2015s11	9	2015s06	AH3N2	2015w18	2015w30
10	2015/20165	15/10/15	2016s04	2016s14	11	2016s11	B	2016w18	2016w30

* Established by an official communiqué of the national health insurance fund and the ministry of health

**Weeks of maximum incidence

***Serotypes: Dominant or co-dominant virus types and subtypes circulating during the season. This information is based on variable numbers of samples and uses virus detection techniques of different sensitivity. Moreover, several definitions of co-dominance may have been used, and this information is provided as a rough guide.

For peer review only

Table 2. List of ICD-10-CA codes included in the administrative case definition of influenza-like illness*.

Diagnosis*	ICD-10 code
Cold	J00
Sinusitis	J01 or J32
Pharyngitis	J02
Laryngitis, tracheitis, or laryngotracheitis	J04
Upper respiratory tract infection	J06.8 or J06.9
Influenza	J09 J10 or J11
Viral pneumonia	J12
Acute bronchitis or bronchitis NOS or obstructive bronchitis	J20 or J40 or J44.8
Bronchiolitis	J21
Acute lower respiratory tract infection, not otherwise specified	J22
COPD with acute lower respiratory tract infection (includes pneumonia)	J44.0
COPD with acute exacerbation	J441
Cough	R05
Pleurisy	R09.1
Pneumonia	J13 or J14 or J15 or J16
Pneumonia, organism unspecified	J18

*Developed using pilot data from emergency departments in Edmonton, Alberta. ICD codes were extracted from randomly selected cases comprising 15% of all emergency department visits with a main ambulatory care diagnosis of influenza.

For peer review only

Table 3. Classification of diseases that can be covered by chronic disease status, in four risk categories, according to the recommendations of the high council for public health (HCSP) with the number of the corresponding condition or disease).

1-RESPIRATORY DISEASES
9 Severe forms of neurological or muscular conditions (including myopathy), severe epilepsy;
14 Severe chronic respiratory failure;
18 Cystic fibrosis;
20 Paraplegia;
25 Multiple sclerosis;
29 Active tuberculosis;
2-CARDIOVASCULAR DISEASES
1) Disabling stroke;
3 Chronic arterial disease with ischemic events;
5) Severe heart failure, serious arrhythmia, severe heart valve defects, severe congenital heart disease; severe congenital heart disease;
13 Coronary disease;
3-KIDNEY FAILURE
19) Severe chronic nephropathy and primary nephrotic syndrome;
4-NEOPLASMS and/or IMMUNE DEFICIENCIES
2 Bone marrow failure and other chronic cytopoenia;
6 Chronic active liver disease and cirrhosis;
7 severe primary immunodeficiency, requiring prolonged treatment, HIV infection;
21 Polyarteritis nodosa, acute disseminated lupus erythematosus, progressive generalised scleroderma;
22 Severe progressive rheumatoid arthritis;
28 Effects of organ transplantation;
30 Malignant tumours, malignant lymphatic or haematopoietic condition.

For peer review only

STROBE 2007 (v4) Statement—Checklist of items that should be included in reports of *case-control studies*

Section/Topic	Item #	Recommendation	Reported on page #
**Title and abstract**	1	(*a*) Indicate the study's design with a commonly used term in the title or the abstract	1
		(*b*) Provide in the abstract an informative and balanced summary of what was done and what was found	2
**Introduction**			
Background/rationale	2	Explain the scientific background and rationale for the investigation being reported	4
Objectives	3	State specific objectives, including any prespecified hypotheses	4
**Methods**			
Study design	4	Present key elements of study design early in the paper	5
Setting	5	Describe the setting, locations, and relevant dates, including periods of recruitment, exposure, follow-up, and data collection	5
Participants	6	(*a*) Give the eligibility criteria, and the sources and methods of case ascertainment and control selection. Give the rationale for the choice of cases and controls	6
		(*b*) For matched studies, give matching criteria and the number of controls per case	6
Variables	7	Clearly define all outcomes, exposures, predictors, potential confounders, and effect modifiers. Give diagnostic criteria, if applicable	8-10
Data sources/ measurement	8*	For each variable of interest, give sources of data and details of methods of assessment (measurement). Describe comparability of assessment methods if there is more than one group	8-10
Bias	9	Describe any efforts to address potential sources of bias	10-11
Study size	10	Explain how the study size was arrived at	Fixed study size
Quantitative variables	11	Explain how quantitative variables were handled in the analyses. If applicable, describe which groupings were chosen and why	8-10
Statistical methods	12	(*a*) Describe all statistical methods, including those used to control for confounding	7
		(*b*) Describe any methods used to examine subgroups and interactions	8
		(*c*) Explain how missing data were addressed	No missing data
		(*d*) If applicable, explain how matching of cases and controls was addressed	6
		(*e*) Describe any sensitivity analyses	6
**Results**			

For peer review only

Participants	13*	(a) Report numbers of individuals at each stage of study—eg numbers potentially eligible, examined for eligibility, confirmed eligible, included in the study, completing follow-up, and analysed	5
		(b) Give reasons for non-participation at each stage	No non-participant
		(c) Consider use of a flow diagram	After the analyzes
Descriptive data	14*	(a) Give characteristics of study participants (eg demographic, clinical, social) and information on exposures and potential confounders	After the analyses
		(b) Indicate number of participants with missing data for each variable of interest	After the analyses
Outcome data	15*	Report numbers in each exposure category, or summary measures of exposure	After the analyses
Main results	16	(*a*) Give unadjusted estimates and, if applicable, confounder-adjusted estimates and their precision (eg, 95% confidence interval). Make clear which confounders were adjusted for and why they were included	7
		(*b*) Report category boundaries when continuous variables were categorized	8-10
		(*c*) If relevant, consider translating estimates of relative risk into absolute risk for a meaningful time period	6
Other analyses	17	Report other analyses done—eg analyses of subgroups and interactions, and sensitivity analyses	6
**Discussion**			
Key results	18	Summarise key results with reference to study objectives	No
Limitations	19	Discuss limitations of the study, taking into account sources of potential bias or imprecision. Discuss both direction and magnitude of any potential bias	10-11
Interpretation	20	Give a cautious overall interpretation of results considering objectives, limitations, multiplicity of analyses, results from similar studies, and other relevant evidence	15
Generalisability	21	Discuss the generalisability (external validity) of the study results	No
**Other information**			
Funding	22	Give the source of funding and the role of the funders for the present study and, if applicable, for the original study on which the present article is based	15

*Give information separately for cases and controls in case-control studies and, if applicable, for exposed and unexposed groups in cohort and cross-sectional studies.

For peer review only

Note: An Explanation and Elaboration article discusses each checklist item and gives methodological background and published examples of transparent reporting. The STROBE checklist is best used in conjunction with this article (freely available on the Web sites of PLoS Medicine at http://www.plosmedicine.org/, Annals of Internal Medicine at http://www.annals.org/, and Epidemiology at http://www.epidem.com/). Information on the STROBE Initiative is available at www.strobe-statement.org.

For peer review only

Effectiveness of seasonal influenza vaccination in patients with diabetes: protocol for a nested case-control study

Journal:	*BMJ Open*
Manuscript ID	bmjopen-2017-016023.R1
Article Type:	Protocol
Date Submitted by the Author:	26-Apr-2017
Complete List of Authors:	Casanova, Ludovic; INSERM, , , Marseille, France, UMR912 "Economics and Social Sciences Applied to Health & Analysis of Medical Information" (SESSTIM)<br>Cortaredona, Sebastien; INSERM, UMR912 "Economics and Social Sciences Applied to Health & Analysis of Medical Information" (SESSTIM), ; ORS PACA, Southeastern Health Regional Observatory,<br>Gaudart, Jean; SESSTIM, INSERM, UMR912 "Economics and Social Sciences Applied to Health & Analysis of Medical Information" (SESSTIM) Marseille, FR; Institut de recherche pour le developpement<br>Launay, Odile; Inserm, F-CRIN, Innovative clinical research network in vaccinology (I-REIVAC), Paris, France; Paris Descartes University, Sorbonne Paris Cité, Assistance Publique Hôpitaux de Paris, Cochin<br>Vanhems, Philippe; Université de Lyon; Université Lyon 1; CNRS UMR 5558, Biométrie et Biologie Evolutive, Epidémiologie et Santé Publique, Lyon, France, Hospices Civils de Lyon, Hôpital Edouard Herriot, Service d'Hygiène, Epidémiologie et Prévention, Lyon, France<br>Villani, Patrick; SESSTIM, INSERM, UMR912 "Economics and Social Sciences Applied to Health & Analysis of Medical Information" (SESSTIM), F-13385, Marseille, France; Observatoire Regional de la Sante Provence-Alpes-Cote d'Azur<br>Verger, Pierre; Aix Marseille Université , SESSTIM UMR 912, Inserm IRD; ORS PACA, Observatoire Régional de la Santé Provence-Alpes-Côte d'Azur
**Primary Subject Heading**:	Infectious diseases
Secondary Subject Heading:	Diabetes and endocrinology, Public health, Research methods, Evidence based practice
Keywords:	General diabetes < DIABETES & ENDOCRINOLOGY, EPIDEMIOLOGY, Public health < INFECTIOUS DISEASES, PUBLIC HEALTH, VIROLOGY

SCHOLARONE™
Manuscripts

TITLE PAGE

Title:

Effectiveness of seasonal influenza vaccination in patients with diabetes: protocol for a nested case-control study

Authors :

Ludovic Casanova[1,2,3,4], Sébastien Cortaredona[1,2,4], Jean Gaudart[1,4], Odile Launay[5,6], Philippe Vanhems[5,7], Patrick Villani[1,2,4], Pierre Verger[1,2,4,5]

Affiliations:

1 INSERM, UMR912 "Economics and Social Sciences Applied to Health & Analysis of Medical Information" (SESSTIM), F-13385, Marseille, France

2 ORS PACA, Southeastern Health Regional Observatory, 13006, Marseille, France

3 Aix Marseille University, Department of general practice, Marseille, France

4 Aix Marseille University, UMR_S912 (SESSTIM), IR(F-13385, Marseille, France

5 Inserm, F-CRIN, Innovative clinical research network in vaccinology (I-REIVAC), Paris, France

6 Paris Descartes University, Sorbonne Paris Cité, Assistance Publique Hôpitaux de Paris, Cochin

7 Infection Control and Epidemiology Unit, Edouard Herriot Hospital, Hospices Civils de Lyon, Lyon, France

Corresponding author:

Ludovic Casanova, Observatoire régional de la santé Provence-Alpes-Côte d'Azur, 23 rue Stanislas Torrents, 13006 Marseille, France (Phone: +33 4 91 59 89 14, Fax: +33 4 91 59 89 18)

Email: ludovic.casanova@inserm.fr

The dates of the study : data necessary for the study will be available in June 2017.

Manuscrit:

4393 words (excluding references, tables, and abstract) / 3 tables

For peer review only

For peer review only - http://bmjopen.bmj.com/site/about/guidelines.xhtml

ABSTRACT

Introduction: Seasonal influenza vaccination (SIV) is recommended for people with diabetes, but its effectiveness has not been demonstrated. All of the available studies are observational and marred with the *healthy vaccinee bias*, that is, bias resulting from the generally better health behaviours practised by people who choose to be vaccinated against influenza, compared with those who do not. This protocol is intended to study the effectiveness of SIV in people with treated diabetes and simultaneously to control for bias.

Methods and analyses: This case-control study is nested in a historical cohort and is designed to study vaccine effectiveness (VE) assessed by morbidity, mortality, and anti-infective drug use. The cohort will comprise a representative sample of health insurance beneficiaries in France and will cover 10 consecutive epidemic seasons. It will include all patients reimbursed three separate times for drugs to treat diabetes. The first study of VE will use reasons for hospitalisation as the primary endpoint, and the second with the use of neuraminidase inhibitors and of antibiotics as the endpoints. A case will be defined as any person in the cohort reaching any endpoint at a given date. The case patient will be matched with the largest possible number of controls (individuals not reaching the endpoint by this date) according to the propensity score method with an optimal caliper width. A conditional logistic model will be used to estimate odds ratios to take into account both the matching and the repetition of measurements. The model will be applied separately during and outside of epidemic periods to estimate the residual confounding.

Ethics and dissemination: The study has been approved by the French Commission on Individual Data Protection and Public Liberties (ref: AT/CPZ/SVT/JB/DP/CR05222O). The study's findings will be published in peer-reviewed journals and disseminated at international conferences and through social media.

Abbreviations:

vaccine effectiveness (VE)

seasonal influenza vaccination (SIV)

general beneficiary sample (EGB)

10^{e} International Classification of Diseases (ICD-10)

relative risk (RR)

neuraminidase inhibitors (NAIs)

anatomical, treatment and chemical (ATC) classification

Keywords :

influenza vaccines

diabetes mellitus

nested case-control

propensity score ; protocol

For peer review only - http://bmjopen.bmj.com/site/about/guidelines.xhtml

Strengths and limitations of this study

- This study will generate new evidence to strengthen public policy recommendations for vaccination of diabetics, especially among adults of working-age.
- This is the first rigorous assessment of the effectiveness of influenza vaccination using the French National Health Insurance System database permitting analysis of about 40 000 patients over a ten year period
- This study explicitly accounts for healthy vaccinee bias that arises in observational studies where those patients who are more likely to be vaccinated are also more likely to prioritise other areas of their health. This bias may result in overestimation of vaccine effectiveness. In our study, we hypothesise that matching cases and controls by their propensity for other preventive behaviours may reduce this bias.
- The analysis includes a comparison of a non-epidemic control period to assess the presence of residual confounding associated with this healthy vaccinee bias.
- As pneumococcal pneumonia is a frequent complication of influenza, and this vaccine is recommended for most diabetics at risk of influenza, this study also considers the pneumococcal vaccination status for the five years before an event.
- By contrast to earlier studies, this study will use a more specific outcome measure for influenza.

For peer review only - http://bmjopen.bmj.com/site/about/guidelines.xhtml

INTRODUCTION

Seasonal influenza is an important cause of morbidity and mortality.[1] Its incidence is higher in children and young adults, but the morbidity and mortality it causes increase with age and in the presence of some chronic diseases, including diabetes.[2,3] The World Health Organization (WHO) recommends seasonal influenza vaccination (SIV) for patients with diabetes[4] because of their high risk of developing severe complications linked to this infection.[2,5] Most studies report that patients with diabetes and healthy patients have equivalent humoral responses to SIV.[6–8] Nonetheless, because influenza antibody titres do not correlate perfectly with clinical protection,[9] vaccine effectiveness (VE) studies are necessary. Recently two systematic reviews observed a lack of evidence of SIV effectiveness in patients with diabetes and concluded that observational studies are urgently needed to improve the methodological flaws of earlier studies.[10,11]

The measurement of SIV effectiveness in at-risk populations is subject to several methodological difficulties. Randomised clinical trials are not used to measure the effectiveness of recommended vaccines in the general population for ethical reasons; they have long been used and their recommendation indicates their effectiveness.[12] Seven observational studies[13–19] have been performed specifically in patients with diabetes to measure this effectiveness, two[13,15] in patients younger than 65 years. For reasons of feasibility and cost, none of these studies diagnosed influenza directly, by either PCR or serology. Instead they used indirect outcome measures, such as all-cause hospitalisation or all-cause death: this type of unspecific endpoint provides greater statistical power but also increases classification biases. Moreover, five of these studies did not take history of pneumococcal vaccination into account although influenza and pneumococcal pulmonary infection can present very similar clinical pictures, and vaccinations against these two diseases have common determinants of adherence.[19,20] Finally, all seven studies are subject to the *healthy vaccinee bias:*[21,22] this bias results from the fact that people who are vaccinated against influenza are, on the whole, more attentive to their health, visit their physicians more often, and probably have better controlled diabetes than unvaccinated individuals. These confounding factors may explain the lower levels of morbidity and mortality observed in vaccinated individuals, independently of the vaccine's protective effect. Accordingly, vaccinated patients have lower rates of morbidity and mortality during non-epidemic periods (that is, seasons during which the influenza virus is not circulating; several authors describe these lower rates as residual confounding.[10,11]

This article presents the protocol of an observational study which has as its principal objective the assessment of SIV effectiveness in patients with treated type 1 and type 2 diabetes while attempting to minimize the preceding methodological limitations. The protocol conducts two nested case-control studies within the very large cohort of beneficiaries of the principal health insurance fund in France. The first study will analyse VE by the reduction in hospitalisations for clinical pictures suggestive of influenza (composite criterion) and for all-cause hospitalisation, and the second by the reduction in outpatient use of neuraminidase-inhibiting antiviral agents (NAIs) and of the antibiotics indicated for treatment of post-influenza lung diseases.

For peer review only

For peer review only - http://bmjopen.bmj.com/site/about/guidelines.xhtml

METHODS AND ANALYSES

Database

The study will be performed by analysing reimbursement data from the French national health insurance fund. Data will be anonymously extracted from the fund's permanent sample of beneficiaries (“Echantillon Généraliste des Bénéficiaires”, EGB) database. The EGB, created in 2003, is a permanent representative sample of persons affiliated to the French health insurance system, a cohort of beneficiaries for whom information on health-care utilisation is conserved for 20 years. These data can thus be used to assess longitudinal utilisation patterns for research purposes. It is obtained by a 1/97th national random sampling with stratification for age and sex as described in more detail previously.[23] The identification of persons included in the sample is protected by a two-level cryptographic anonymisation process. The EGB is obtained from the reimbursement databases of the major public health insurance funds (salaried workers), which record information on the health-care utilisation of nearly 85% of the general population in France. On 1 January, 2016, the EGB database included 675 906 individuals for whom it contains the following information: age, sex, all reimbursements for medical expenses for outpatient care, and their “chronic disease status“ with its diagnostic codes according to the 10th revision of the International Classification of Diseases (ICD-10). “Chronic disease status” is attributed to people with specific and expensive chronic diseases defined by the French health insurance system and makes them eligible for 100% reimbursement of the costs of their treatment as described in more detail previously.[24] Since January 2006, the EGB has also provided access to all episodes of hospitalisation in public and private hospitals and to their associated discharge diagnoses, coded according to ICD-10.

Definition of the study cohort

This historical cohort of people treated for diabetes will be followed from 12 October, 2006 (date that the 2006-2007 campaign vaccination started), through 9 October, 2016 (day before the start of the 2016-2017 campaign vaccination). Patients will be included only if they were in the database for at least 3 years before their follow-up begins. People treated for diabetes will be identified according to the following criteria, previously used in epidemiologic studies: they are listed in the databases as having received at least three deliveries of oral diabetes drugs or insulin on three distinct dates in the 12 months before inclusion.[25] For patients to whom oral diabetes drugs were dispensed in the year before the start of follow-up (prevalent diabetes at inclusion), the inclusion date will be the study start date. For new cases of treated diabetes (incident cases of treated diabetes during the study period), the date of inclusion will be the date of the first delivery of diabetes drugs. Patients who change insurance funds or die during follow-up will be censored.

Years of follow-up

For peer review only - http://bmjopen.bmj.com/site/about/guidelines.xhtml

The maximum duration of follow-up will be 10 years. A year of follow-up will begin on the first day of the SIV campaign of the year *n* and will end the day preceding the start of the campaign for the year *n*+1. The French Ministry of Defence sets the dates that SIV campaigns begin each year (table 1). The covariables will be assessed at the beginning of each year of follow-up (baseline variables) and considered stable through the end of that year.

SIV status

During each year of follow-up, SIV status will be defined as positive 8 days after the day the vaccine was dispensed to the patient at the pharmacy (the minimum interval before protective antibodies appear[26–28]). This delivery will be identified by its code in the Anatomical, Treatment and Chemical classification for influenza vaccines (ATC Code J07BBxx). We hypothesise that patients will be vaccinated the day they purchase the vaccine at the pharmacy. A sensitivity analysis will test different intervals between the vaccine purchase date and the vaccination date to verify the impact of this hypothesis on the results.

Cohort-nested case-control study

A case will be defined as any person in the cohort presenting any endpoint at a given date. For each case, the controls will be defined as all patients not presenting that endpoint up to the date it occurred for the case. A patient can be chosen as a control numerous times. Any patient who presents an endpoint during a given year becomes a case and is thus excluded from the control pool until the beginning of the next year. Controls will be matched to cases according to the propensity score method.[29] The propensity score is a one-dimensional summary of all of the matching variables and will furnish an estimate of the probability of being vaccinated as a function of several specific variables. It will be calculated by logistic regression with vaccination as the dependent variable. The model's explanatory variables will be: the date of cohort entry, to control for variations over time (+/- 6 months), age, sex, adherence to secondary prevention examinations for diabetes follow-up, visits to general practitioners, history of pneumococcal vaccination, comorbidities, diabetes severity, hospitalisation in the previous year, and SIV history. In the most common implementation of propensity-score matching, pairs of cases and controls are formed when their propensity scores differ by a pre-specified maximum amount. To minimise means or risk differences, we will match for the logit of the propensity score, using calipers of width equal to 0.2 of the standard deviation of the logit of the propensity score [30]. The adjusted odds ratio from this conditional logistic regression will be corrected to approach a relative risk (RR) according to the method of Zhang and Yu [31]. The following formula will be used to calculate the VE:

$$VE\ (\%) = (1 - RR) * 100$$

First study: endpoints based on hospitalisation and its causes

The principal endpoint will be hospitalisation for a clinical picture suggestive of influenza or an indirect complication (table 2). Specifically, it will be the composite criterion of the ICD-10 codes used by Lau

For peer review only - http://bmjopen.bmj.com/site/about/guidelines.xhtml

et al. in 2013 to study VE.[15] It is based on the reasons for hospitalisation of patients referred by private-practice physicians to hospital emergency departments for influenza. This outcome is in line with the frequency of clinical complications associated with influenza. For example in England, among 141,293 patients with diagnosed influenza, 9.5% presented clinical complications in the 30 days after diagnosis: 1.5% bronchitis, 0.4% lung disease, 5.5% unspecified upper respiratory tract infection.[32] An Italian study [33] reported a higher complication rate (30%,) with the complications distributed similarly. Some of the clinical complications included by Lau et al (colds, laryngitis and coughs) are too non-specific; we therefore did not include them in the algorithm. The date of the onset of the criterion will be the first day of hospitalisation. The secondary endpoint will be death or hospitalisation from all causes combined, excluding planned admissions.

Second study: Endpoints based on outpatient prescriptions

The principal endpoint will be the dispensing by a private pharmacy of an NAI (ATC Code: J05AH, oseltamivir or zanamivir). NAIs are systemic antiviral treatments used to cure seasonal influenza in the 48 hours after its first symptoms in individuals at risk of complications, including patients with diabetes, but is prescribed on an outpatient basis to only approximately 25% of patients for whom it is indicated.[34] This endpoint makes it possible to identify a portion of the influenza cases receiving outpatient care.

The secondary endpoint will be the delivery in a private pharmacy of one of the antibiotic treatment recommended [35] as a first- or second-line treatment for cases of acute, community-acquired post-influenza lung diseases: amoxicillin, amoxicillin/clavulanic acid (ATC Code: J01CR, excluding the following compounds with the same code: ticarcillin/clavulanic acid, ampicillin/sulbactam and piperacillin/tazobactam), ceftriaxone (ATC Code: J01DD04), pristinamycin (ATC Code: J01FG01), levofloxacin (ATC Code: S01AE05), and telithromycin (ATC Code: J01FA15). These recommendations did not change during the study period.

Statistical analyses

To take into account the variable number of controls per case, the descriptive statistics will be weighted by the inverse of the number of controls in those analyses. As recommended for the nested case-control study design,[36] a conditional logistic model will be used to calculate the odds ratio to take into account both the matching of individuals in the analysis and the repetition of measurements. Recommendations for use of propensity score matching suggest adjusting for the propensity score, because matching plus adjustment reduces the previous imbalance between two cases and controls more effectively than matching alone.[37] Adjusting for the propensity score thus improves the estimate of the intervention effect.

This model will be applied separately during epidemic periods and non-epidemic periods to estimate possible residual confounding due to the healthy vaccinee bias. Each year, the epidemic period will be defined as the period during which the incidence of influenza-like illnesses (ILIs) exceeds the epidemic thresholds defined from influenza surveillance data in France (table 1).[38–40] The non-

For peer review only - http://bmjopen.bmj.com/site/about/guidelines.xhtml

epidemic periods will be those during which there is no recorded circulation of the virus: 15 May to 31 July each year since 1985 (websenti.u707.jussieu.fr).

Moreover, the analyses will be stratified for age: they will be performed separately for patients aged 65 years or older and for those younger than 65 years. SIV is doubly indicated for patients with diabetes aged 65 years or older — for both their age and their chronic condition. Vaccination coverage of people with diabetes younger than 65 years is lower than that of older patients,[20] and clear VE data lack for this younger age group, who account for nearly half the patients with diabetes treated pharmacologically in France.[41]

Mismatch between SIV and circulating viruses

The intensity of epidemics varies from year to year due to antigenic modifications of influenza virus strains that can result in a poor match between the circulating strains and those contained in the vaccine and thus result in variations in SIV effectiveness. For instance, mismatches for the A(H3N2) virus during the 2012-2013 and 2014-2015 seasons may explain the poor vaccine effectiveness observed among the elderly during those seasons. [42] The use of data from 10 epidemic seasons will allow us to construct a "step by step" sensitivity analysis, by excluding years in decreasing order of the extent of the mismatch. We hypothesize that the VE should increase if statistical power remains sufficient.

Justification of the variables included in the propensity score calculation

1/ Age and sex

The risks of hospitalisation and influenza-related complications increase with age, while influenza incidence decreases with age. Age will be defined in the following classes: [18-35[; [35-45[; [45-55[; [55-65[; [65-75[; [75-85[; >85. Including age in the calculation of the propensity score enables consideration of the immunosenescence that reduces VE in older subjects, through its impairment of response to new antigens and its reduction in immune memory.[43,44].For the sex, men appear to have better SIV coverage[45] and are vaccinated with greater regularity.[20]

3/ Adherence to examinations for secondary prevention in diabetes follow-up

Adherence to SIV depends, in part, on the behaviour and attitudes of both the patient and the general practitioner in the follow-up of chronic diseases.[46] Patients with diabetes who are vaccinated against influenza are also more adherent in the follow-up of their diabetes than those not vaccinated;[47], this can affect both the control and complications of their diabetes. In people with diabetes, inadequate disease control is associated with 80% of all-cause hospitalisation.[13,16] These points partly explain the healthy vaccinee bias when the VE is calculated from hospital admissions. A proxy variable for adherence with secondary prevention of people with diabetes will be constructed according to a previously published method.[48] This adherence score ranges from 0 to 5 and attributes a value of 1 for each of these examinations performed during the past three years: HbA1C, microalbuminuria, LDL-cholesterol, ocular fundus, podiatrist consultation. This score has been shown to be partially correlated with the frequency of general practitioner visits, but not collinear with it.[49]

For peer review only - http://bmjopen.bmj.com/site/about/guidelines.xhtml

4/ Frequency of visits to a general practitioner in the past year

The probability of vaccination against influenza increases with the number of visits to general practitioners.[20,50] For patients 65 years or older with diabetes, Rodriguez et al[19] found a mean of 14 annual GP consultations among the unvaccinated patients versus 20 among those vaccinated. GP consultations can modify patients' health care pathway by reducing the risk of hospitalisation, for example, by earlier care for influenza (treatment by NAIs in the first 48 first hours of an ILI[34]). At the same time, it enables the best possible diabetes control through more regular monitoring. Preceding studies of VE show that it is preferable to include it as a continuous variable.[13,15]

5/ History of pneumococcal vaccination

Patients with diabetes are at increased risk of invasive pneumococcal infections.[51–53] Adherence to SIV and to pneumococcal vaccination have common determinants,[50] with pneumococcal vaccination coverage is best in patients vaccinated against influenza.[19] Without adjustment for history of this vaccination, SIV effectiveness may be overestimated. The official recommendation of routine pneumococcal vaccination in patients with diabetes dates back only to 2009 in France but such vaccination took place before then. To construct this adjustment variable, we will consider as vaccinated all patients with at least one delivery of the pneumococcal vaccine (*ATC Code*: J07A L01) in the 5 years before the year under consideration (In accordance with french guidelines : period of persistence of the protective antibody level with certainty [54,55]). Pneumococcal vaccination status will be considered positive the day the vaccine is dispensed at the pharmacy. Like influenza vaccination status, pneumococcal vaccination status will not be a baseline value but may vary over a year of follow-up. Because of this variable's importance for potential confounding, an interaction term will be added to the models. Should the latter be significant, an analysis stratified by pneumococcal vaccination will be performed to estimate SIV effectiveness in patients with pneumococcal vaccination within the past 5 years and in those vaccinated either never or not in the past 5 years.

6/ Diabetes severity

The severity of diabetes is simultaneously positively associated with the risk of hospitalisation for infectious causes,[56] including seasonal influenza,[2,57] and with vaccination coverage.[20] It is therefore a confounding factor. Because the EGB databases do not include any individual clinical or laboratory variable to measure diabetes severity directly, it will be approached indirectly by two different variables:

- The intensity of the treatments prescribed: a single oral diabetes medication for mild diabetes, two different such medications for moderate diabetes, and more than two different drugs and/or insulin for severe diabetes;
- Time since diagnosis of diabetes is associated with the risks of influenza complications and of immunodeficiency secondary to chronic hyperglycaemia. A proxy variable for this duration will be the estimated as of 2003 (date of earliest EGB data).

7/ Comorbidities

For peer review only - http://bmjopen.bmj.com/site/about/guidelines.xhtml

Patients with diabetes and at least one comorbidity have both better vaccination coverage[20] and a higher risk of influenza complications: if this "confounding by indication" is not taken into account, it can result in underestimating SIV effectiveness.[22] For each patient, we have diagnoses (coded with ICD-10) associated with potential chronic disease status, in addition to diabetes. To use this variable most productively, we plan to regroup them into four major disease groups (table 3): respiratory diseases, cardiovascular diseases, kidney diseases, and neoplasms and/or immunodeficiencies.

8/ Hospitalisation in the preceding year

Adjustment for this variable is also intended to improve control for confounding by indication. Hospitalisation during the preceding year can result in behavioural changes in subsequent years.[58] We can expect an excess of hospitalisations in vaccinated patients, associated not with influenza complications but rather with fragility related to health status. This variable will be constructed dichotomously and separately for each year of follow-up: no hospitalisation or at least one hospitalisation for any cause (excluding planned hospitalisations) during the year preceding the year of follow-up considered.

9/ History of SIV

Currently, the literature about the effect of repeated SIV is contradictory. A meta-analysis published in 1999 suggested that repeated SIV does not affect its effectiveness.[59] A recent study of vaccinated patients showed a reduction in influenza-positive respiratory infections that was greatest among those not vaccinated for the preceding 5 years.[60] Nonetheless other work has shown that the serologic protection against influenza caused by vaccination may persist from one year to another if the virus does not mutate, [57] as it regularly does in practice. Accordingly, one study observed that mortality fell by 15% more in the group regularly vaccinated against influenza than in the newly vaccinated group.[61] We have therefore chosen to adjust for the presence of SIV during the two years preceding the year under consideration, as earlier studies have done.[13]

Discussion

Strengths and limitations of the study design

The design of a case-control study nested in a cohort is a sampling technique based on the principle of sampling within the group at risk ("risk set sampling") and simplifies consideration of exposures and covariables that vary over time. This design optimises the use of the available data by making it possible for a patient to be, at different times, a control and a case and thus facilitates the analysis of infrequent events. The hospitalisation rate for influenza in patients with a chronic condition is around 2% in France.[62] This design also enables the study of rare endpoints. It should also allow separate analyses of data restricted to the epidemic period and to the non-epidemic season despite the resulting reduction in the number of observations. Another useful point of this design is the improved comparability between cases and controls made possible by propensity score matching, a method producing the lowest mean quadratic error when large numbers of controls are available.[29,63]

For peer review only

Propensity score matching allows the comparison of patients with the same probability of being vaccinated and thus reduces the healthy vaccinee bias.

Several points led us to rule out using a control period before the epidemic season . In another study, VE off-season was greater in the post- rather than the pre-season. The authors deduced from their finding that the period after the epidemic season would be a better marker of residual confounding and vaccination bias.[15]

The interest in matching with a propensity score compared with strict matching is to give more weight to some variables that better explain vaccination behaviour. For example, adherence probably predicts vaccination behaviour better than sex, so that giving the two equal weight would decrease the quality and utility of the matching. However, we have already tested [64] in a previous article that none of the variables that we plan to use in the matching score was likely to explain SIV behaviour either by itself or when considered jointly in a multiple regression: the risk of overmatching remains low. We nonetheless plan to build an analytic strategy by progressively including the matching variables in the propensity score to assess the impact of the matching variables

Strengths and limitations of endpoints related to hospitalisation and its causes

The principal outcome selected does not resolve the lack of specificity encountered in the preceding studies. Among the influenza complications included in our algorithm are unspecific bacterial superinfections that are common to other viral infections. To limit the lack of specificity, we nonetheless have not included deterioration or decompensation of chronic diseases, considered in some earlier studies. These hospitalizations for decompensated chronic disease are probably more influenced by patients' health behaviours and thus probably more subject to the healthy vaccinee bias. We know that our end point is relatively independent of the healthy vaccinee bias and helps to minimise residual confounding. It is therefore probable that we will not observe differences in this endpoint during the non-epidemic season. Inversely the endpoints of death and all-cause hospitalisation are more sensitive to this bias. Among patients with diabetes aged 65 years or older, SIV also appears to reduce all-cause mortality by 38% or more.[10] Nonetheless the excess mortality associated with influenza is estimated at 5 to 10%.[65,66] A substantial portion of the reduction above is therefore linked to unobserved factors. Most studies applying this criterion have found that VE persists during the off-season. This endpoint has been used as a marker of residual confounding; its failure to appear, contrary to earlier studies, will enable us to shed light on how this design affects it.

Strengths and limitations of the endpoints based on outpatient medication use

In patients with diabetes younger than 65 years, the low rate of hospitalisation for ILI makes it difficult to measure VE. The two studies that studied VE with this criterion in this population found no significant effect.[13,15] No study has yet used outpatient NAI use to measure VE, but evidence indicates its relevance. NAIs are a specific treatment for the influenza virus. NAIs can only be purchased on an outpatient basis if prescribed by a doctor. The specificity of this outcome therefore reflects the specificity of doctors' influenza diagnoses of ILI. In epidemic periods, their specificity for this diagnosis is on the order of 60-70%). [67,68] Models of chronological series of influenza activity in

For peer review only - http://bmjopen.bmj.com/site/about/guidelines.xhtml

different countries report strong correlations between the official surveillance of sentinel physicians and pharmacy NAI sales,[69–72] especially for patients of working age. The disadvantages of this endpoint are associated with the multiple other therapeutic indications for NAIs, including: i) post-exposure prophylaxis of seasonal influenza in adult after contact with a clinically diagnosed case in the family and in other exceptional situations; ii) prophylaxis, for both pandemic situations and seasonal influenza during epidemic periods when the circulating viral strains do not match the vaccine. No data describe the proportion of NAIs dispensed for these two indications. The other limitation is related to the lack of specificity of the initial clinical presentation of influenza. Work has shown that primary-care physicians' NAI prescriptions are suboptimal,[73] but no evidence shows that this bias is differential.

Antibiotic prescriptions for influenza complications are the second principal component of outpatient care for influenza accessible in the database. Susceptibility to secondary infection of the lungs and bronchi by bacteria, e.g., Streptococcus pneumoniae and Haemophilus influenzae, seems to result from increased binding of bacteria to the basal membrane of the respiratory epithelium. This may be the result of direct viral damage. [74] Furthermore the neuraminidase activity of influenzae viruses might thereby decrease the viscosity of the mucus and increase adherence of pneumococci. [75] Finally, the risk of bacterial infections in patients with diabetes in the presence of influenza is aggravated by the immunodeficiency caused by chronic hyperglycemia. [76] Numerous studies have shown that seasonal influenza epidemics are strongly associated with increased use of antibiotics, especially aminopenicillins and macrolides. [77] Thus, influenza vaccination should result in a reduction in antibiotic use. This variable has already been used as an indirect outcome for assessing SIV effectiveness: in Ontario (Canada): antibiotic prescriptions fell after the introduction of universal free vaccination against influenza. [78,79]

Strengths and limitations associated with the database

This will be the first study of the effectiveness of seasonal influenza vaccine performed in France from a nationwide healthcare-related administrative database. Nonetheless, these databases do not provide access to laboratory results when laboratory tests are performed, and these tests are not systematically performed. The studies of the VE) of SIV with a direct endpoint measure, such as PCR for influenza from nasopharyngeal swabs, show that indirect outcome measures, such as ILI, can overestimate VE compared with the former (direct outcomes).[80]

Moreover it is probable that the quality of coding varies between hospitals. Given that the EGB sample is national and random, these coding variations are probably not differential. This type of bias is likely to induce underestimation of VE, which should be made up for in part by our study's statistical power.

For peer review only - http://bmjopen.bmj.com/site/about/guidelines.xhtml

FOOTNOTES

Acknowledgments : None

Contributors: L. CASANOVA (MD PhD student) drafted this study protocol under the direction of P. VERGER (MD PhD) and P. VILLANI (MD PhD). S. CORTAREDONA (MD statistician) contributed to the data management and construction of the statistical model. J. GAUDART (MD PhD) provided his expertise for the design of the nested case-control study. P. VANHEMS (MD PhD) and Odile LAUNAY (MD PhD) provided their expertise about vaccination and vaccine effectiveness. All the authors participated in the drafting and revision of the manuscript. All authors were major contributors in interpretation of data and in writing the manuscript. All authors approved the final version and agree to be accountable for the contents and integrity of this manuscript.

Competing interests: None.

Funding: This research received no specific grant from any funding agency in the public, commercial or not-for-profit sectors

Ethics approval: Article 3 of the decree dated 20 June 2005 concerning the implementation of the interregional health insurance information system (updated by the decree dated 11 July 2012) foresaw the establishment of a sample of patients affiliated with the French national health insurance system, to be called the general beneficiary sample (EGB). Data for this sample may be conserved for 20 years after the day's date. This panel is administered by the CNAM-TS and has been approved by the CNIL (CNIL (French Commission on Individual Data Protection and Public Liberties, study registered under number AT/CPZ/SVT/JB/DP/CR05222O of 14 June 2005 and number DP/CR071761 of 28 August 2007. French law does not require authorisation of an ethics committee for the use of routinely collected data by authorised research teams (Official Journal of the Republic of French, 2012). The mixed research unit (AMU/Inserm/IRD) that will supervise this study is so authorised.

For peer review only - http://bmjopen.bmj.com/site/about/guidelines.xhtml

REFERENCES

1 Dawood FS, Iuliano AD, Reed C, *et al.* Estimated global mortality associated with the first 12 months of 2009 pandemic influenza A H1N1 virus circulation: a modelling study. *Lancet Infect Dis* 2012;**12**:687–95. doi:10.1016/S1473-3099(12)70121-4

2 Muller LMAJ, Gorter KJ, Hak E, *et al.* Increased risk of common infections in patients with type 1 and type 2 diabetes mellitus. *Clin Infect Dis* 2005;**41**:281–8. doi:10.1086/431587

3 Isotani H. Use of influenza vaccine in patients with diabetes, particularly those with complications of cardiac or renal disease. *Intern Med* 2000;**39**:862.

4 World Health Organization. Fact sheet on influenza. 2014. WHO. http://www.who.int/mediacentre/factsheets/fs211/en/ (accessed 21 Jul 2015).

5 Mertz D, Kim TH, Johnstone J, *et al.* Populations at risk for severe or complicated influenza illness: systematic review and meta-analysis. *BMJ* 2013;**347**:f5061.

6 Frasca D, Diaz A, Romero M, *et al.* Young and elderly patients with type 2 diabetes have optimal B cell responses to the seasonal influenza vaccine. *Vaccine* 2013;**31**:3603–10. doi:10.1016/j.vaccine.2013.05.003

7 Pozzilli P, Gale EA, Visalli N, *et al.* The immune response to influenza vaccination in diabetic patients. *Diabetologia* 1986;**29**:850–4.

8 el-Madhun AS, Cox RJ, Seime A, *et al.* Systemic and local immune responses after parenteral influenza vaccination in juvenile diabetic patients and healthy controls: results from a pilot study. *Vaccine* 1998;**16**:156–60.

9 Reber A, Katz J. Immunological assessment of influenza vaccines and immune correlates of protection. *Expert Rev Vaccines* 2013;**12**:519–36. doi:10.1586/erv.13.35

10 Remschmidt C, Wichmann O, Harder T. Vaccines for the prevention of seasonal influenza in patients with diabetes: systematic review and meta-analysis. *BMC Med* 2015;**13**:53. doi:10.1186/s12916-015-0295-6

11 Casanova L, Gobin N, Villani P, *et al.* Bias in the measure of the effectiveness of seasonal influenza vaccination among diabetics. *Prim Care Diabetes* Published Online First: 8 June 2016. doi:10.1016/j.pcd.2016.05.005

12 Fiore AE, Uyeki TM, Broder K, *et al.* Prevention and control of influenza with vaccines: recommendations of the Advisory Committee on Immunization Practices (ACIP), 2010. *MMWR Recomm Rep* 2010;**59**:1–62.

13 Akker IL-V den, Verheij TJM, Buskens E, *et al.* Clinical Effectiveness of First and Repeat Influenza Vaccination in Adult and Elderly Diabetic Patients. *Dia Care* 2006;**29**:1771–6. doi:10.2337/dc05-2517

14 Wang I-K, Lin C-L, Chang Y-C, *et al.* Effectiveness of influenza vaccination in elderly diabetic patients: a retrospective cohort study. *Vaccine* 2013;**31**:718–24. doi:10.1016/j.vaccine.2012.11.017

15 Lau D, Eurich DT, Majumdar SR, *et al.* Effectiveness of influenza vaccination in working-age adults with diabetes: a population-based cohort study. *Thorax* 2013;**68**:658–63. doi:10.1136/thoraxjnl-2012-203109

16 Colquhoun AJ, Nicholson KG, Botha JL, *et al.* Effectiveness of influenza vaccine in reducing hospital admissions in people with diabetes. *Epidemiol Infect* 1997;**119**:335–41.

For peer review only - http://bmjopen.bmj.com/site/about/guidelines.xhtml

17 Heymann AD, Shapiro Y, Chodick G, *et al.* Reduced Hospitalizations and Death Associated With Influenza Vaccination Among Patients With and Without Diabetes. *Dia Care* 2004;**27**:2581–4. doi:10.2337/diacare.27.11.2581

18 Hak E, Nordin J, Wei F, *et al.* Influence of high-risk medical conditions on the effectiveness of influenza vaccination among elderly members of 3 large managed-care organizations. *Clin Infect Dis* 2002;**35**:370–7. doi:10.1086/341403

19 Rodriguez-Blanco T, Vila-Corcoles A, de Diego C, *et al.* Relationship between annual influenza vaccination and winter mortality in diabetic people over 65 years. *Hum Vaccin Immunother* 2012;**8**:363–70. doi:10.4161/hv.18548

20 Verger P, Cortaredona S, Pulcini C, *et al.* Characteristics of patients and physicians correlated with regular influenza vaccination in patients treated for type 2 diabetes: a follow-up study from 2008 to 2011 in southeastern France. *Clinical Microbiology and Infection*;**0**. doi:10.1016/j.cmi.2015.06.017

21 Hak E, Verheij TJM, Grobbee DE, *et al.* Confounding by indication in non-experimental evaluation of vaccine effectiveness: the example of prevention of influenza complications. *J Epidemiol Community Health* 2002;**56**:951–5. doi:10.1136/jech.56.12.951

22 Remschmidt C, Wichmann O, Harder T. Frequency and impact of confounding by indication and healthy vaccinee bias in observational studies assessing influenza vaccine effectiveness: a systematic review. *BMC Infect Dis* 2015;**15**:429. doi:10.1186/s12879-015-1154-y

23 Tuppin P, de Roquefeuil L, Weill A, *et al.* French national health insurance information system and the permanent beneficiaries sample. *Rev Epidemiol Sante Publique* 2010;**58**:286–90. doi:10.1016/j.respe.2010.04.005

24 Cortaredona S, Pambrun E, Verdoux H, *et al.* Comparison of pharmacy-based and diagnosis-based comorbidity measures from medical administrative data. *Pharmacoepidemiol Drug Saf* Published Online First: 2 December 2016. doi:10.1002/pds.4146

25 Tiv M, Viel J-F, Mauny F, *et al.* Medication adherence in type 2 diabetes: the ENTRED study 2007, a French Population-Based Study. *PLoS ONE* 2012;**7**:e32412. doi:10.1371/journal.pone.0032412

26 Halliley JL, Kyu S, Kobie JJ, *et al.* Peak frequencies of circulating human influenza-specific antibody secreting cells correlate with serum antibody response after immunization. *Vaccine* 2010;**28**:3582–7. doi:10.1016/j.vaccine.2010.02.088

27 Cox RJ, Brokstad KA, Zuckerman MA, *et al.* An early humoral immune response in peripheral blood following parenteral inactivated influenza vaccination. *Vaccine* 1994;**12**:993–9.

28 Sasaki S, He X-S, Holmes TH, *et al.* Influence of prior influenza vaccination on antibody and B-cell responses. *PLoS ONE* 2008;**3**:e2975. doi:10.1371/journal.pone.0002975

29 Garrido MM, Kelley AS, Paris J, *et al.* Methods for Constructing and Assessing Propensity Scores. *Health Serv Res* 2014;**49**:1701–20. doi:10.1111/1475-6773.12182

30 Austin PC. Optimal caliper widths for propensity-score matching when estimating differences in means and differences in proportions in observational studies. *Pharm Stat* 2011;**10**:150–61. doi:10.1002/pst.433

31 Zhang J, Yu KF. What's the relative risk? A method of correcting the odds ratio in cohort studies of common outcomes. *JAMA* 1998;**280**:1690–1.

32 Meier CR, Napalkov PN, Wegmüller Y, *et al.* Population-Based Study on Incidence, Risk Factors, Clinical Complications and Drug Utilisation Associated with Influenza in the United Kingdom. *EJCMID* 2000;**19**:834–42. doi:10.1007/s100960000376

For peer review only - http://bmjopen.bmj.com/site/about/guidelines.xhtml

33 Sessa A, Costa B, Bamfi F, *et al.* The incidence, natural history and associated outcomes of influenza-like illness and clinical influenza in Italy. *Fam Pract* 2001;**18**:629–34.

34 Dobson J, Whitley RJ, Pocock S, *et al.* Oseltamivir treatment for influenza in adults: a meta-analysis of randomised controlled trials. *The Lancet* 2015;**385**:1729–37. doi:10.1016/S0140-6736(14)62449-1

35 AFSSAPS. Antibiotics in lower respiratory tract infections in adults and children. French Guidelines. October 2005.

36 Kim MY, Zeleniuch-Jacquotte A. Correcting for measurement error in the analysis of case-control data with repeated measurements of exposure. *Am J Epidemiol* 1997;**145**:1003–10.

37 Caliendo M, Kopeinig S. Some Practical Guidance for the Implementation of Propensity Score Matching. *Journal of Economic Surveys* 2008;**22**:31–72. doi:10.1111/j.1467-6419.2007.00527.x

38 Costagliola D, Flahault A, Galinec D, *et al.* A routine tool for detection and assessment of epidemics of influenza-like syndromes in France. *Am J Public Health* 1991;**81**:97–9.

39 Serfling RE. Methods for current statistical analysis of excess pneumonia-influenza deaths. *Public Health Rep* 1963;**78**:494–506.

40 Stroup DF, Williamson GD, Herndon JL, *et al.* Detection of aberrations in the occurrence of notifiable diseases surveillance data. *Stat Med* 1989;**8**:323-329-332.

41 Fosse-Edorh S, Fagot-Campagna A, Detournay B, *et al.* Impact of socio-economic position on health and quality of care in adults with Type 2 diabetes in France: the Entred 2007 study. *Diabet Med* Published Online First: 17 April 2015. doi:10.1111/dme.12783

42 Centers for Disease Control and Prevention (CDC). Interim adjusted estimates of seasonal influenza vaccine effectiveness - United States, February 2013. *MMWR Morb Mortal Wkly Rep* 2013;**62**:119–23.

43 Goronzy JJ, Weyand CM. Understanding immunosenescence to improve responses to vaccines. *Nat Immunol* 2013;**14**:428–36. doi:10.1038/ni.2588

44 Lang PO, Govind S, Bokum AT, *et al.* Immune senescence and vaccination in the elderly. *Curr Top Med Chem* 2013;**13**:2541–50.

45 Jimenez-Trujillo I, López-de Andrés A, Hernández-Barrera V, *et al.* Influenza vaccination coverage rates among diabetes sufferers, predictors of adherence and time trends from 2003 to 2010 in Spain. *Hum Vaccin Immunother* 2013;**9**:1326–32. doi:10.4161/hv.23926

46 van Essen GA, Kuyvenhoven MM, de Melker RA. Compliance with influenza vaccination. Its relation with epidemiologic and sociopsychological factors. *Arch Fam Med* 1997;**6**:157–162; discussion 163.

47 Achtymichuk KA, Johnson JA, Al Sayah F, *et al.* Characteristics and health behaviors of diabetic patients receiving influenza vaccination. *Vaccine* 2015;**33**:3549–55. doi:10.1016/j.vaccine.2015.05.047

48 Gregg EW, Karter AJ, Gerzoff RB, *et al.* Characteristics of insured patients with persistent gaps in diabetes care services: the Translating Research into Action for Diabetes (TRIAD) study. *Med Care* 2010;**48**:31–7. doi:10.1097/MLR.0b013e3181bd4783

49 Ricci P, Chantry M, Detournay B. Coûts des soins remboursés par l'Assurance maladie aux personnes traitées pour diabète : Études Entred 2001 et 2007. *BEH n°42-43* 2009;:464–8.

50 Clancy U, Moran I, Tuthill A. Prevalence and predictors of influenza and pneumococcal vaccine uptake in patients with diabetes. *Ir Med J* 2012;**105**:298–300.

For peer review only - http://bmjopen.bmj.com/site/about/guidelines.xhtml

51 Thomsen RW, Hundborg HH, Lervang H-H, *et al.* Risk of community-acquired pneumococcal bacteremia in patients with diabetes: a population-based case-control study. *Diabetes Care* 2004;**27**:1143–7.

52 Klemets P, Lyytikäinen O, Ruutu P, *et al.* Risk of invasive pneumococcal infections among working age adults with asthma. *Thorax* 2010;**65**:698–702. doi:10.1136/thx.2009.132670

53 Valdez R, Narayan KM, Geiss LS, *et al.* Impact of diabetes mellitus on mortality associated with pneumonia and influenza among non-Hispanic black and white US adults. *Am J Public Health* 1999;**89**:1715–21.

54 Musher DM, Manof SB, Liss C, *et al.* Safety and antibody response, including antibody persistence for 5 years, after primary vaccination or revaccination with pneumococcal polysaccharide vaccine in middle-aged and older adults. *J Infect Dis* 2010;**201**:516–24. doi:10.1086/649839

55 Fisman DN, Abrutyn E, Spaude KA, *et al.* Prior pneumococcal vaccination is associated with reduced death, complications, and length of stay among hospitalized adults with community-acquired pneumonia. *Clin Infect Dis* 2006;**42**:1093–101. doi:10.1086/501354

56 Bertoni AG, Saydah S, Brancati FL. Diabetes and the risk of infection-related mortality in the U.S. *Diabetes Care* 2001;**24**:1044–9.

57 Peleg AY, Weerarathna T, McCarthy JS, *et al.* Common infections in diabetes: pathogenesis, management and relationship to glycaemic control. *Diabetes Metab Res Rev* 2007;**23**:3–13. doi:10.1002/dmrr.682

58 Hottes TS, Skowronski DM, Hiebert B, *et al.* Influenza vaccine effectiveness in the elderly based on administrative databases: change in immunization habit as a marker for bias. *PLoS ONE* 2011;**6**:e22618. doi:10.1371/journal.pone.0022618

59 Beyer WE, de Bruijn IA, Palache AM, *et al.* Protection against influenza after annually repeated vaccination: a meta-analysis of serologic and field studies. *Arch Intern Med* 1999;**159**:182–8.

60 McLean HQ, Thompson MG, Sundaram ME, *et al.* Impact of repeated vaccination on vaccine effectiveness against influenza A(H3N2) and B during 8 seasons. *Clin Infect Dis* 2014;**59**:1375–85. doi:10.1093/cid/ciu680

61 Voordouw ACG, Sturkenboom MCJM, Dieleman JP, *et al.* Annual Revaccination Against Influenza and Mortality Risk in Community-Dwelling Elderly Persons. *JAMA November 3, 2004* 2004;**292**:2089–95.

62 Groupe de Travail. [Taking care of influenza in the context of a pandemic in 2005. Full text]. *Med Mal Infect* 2005;**35 Suppl 4**:S245-273.

63 Kwiatkowski F, Slim K, Verrelle P, *et al.* [Propensity score: interest and limits]. *Bull Cancer* 2007;**94**:680–6.

64 Verger P, Cortaredona S, Pulcini C, *et al.* Characteristics of patients and physicians correlated with regular influenza vaccination in patients treated for type 2 diabetes: a follow-up study from 2008 to 2011 in southeastern France. *Clin Microbiol Infect* 2015;**21**:930.e1-9. doi:10.1016/j.cmi.2015.06.017

65 Horcajada JP, Pumarola T, Martínez JA, *et al.* A nosocomial outbreak of influenza during a period without influenza epidemic activity. *Eur Respir J* 2003;**21**:303–7.

66 Berg HF, Van Gendt J, Rimmelzwaan GF, *et al.* Nosocomial influenza infection among post-influenza-vaccinated patients with severe pulmonary diseases. *J Infect* 2003;**46**:129–32.

For peer review only

For peer review only - http://bmjopen.bmj.com/site/about/guidelines.xhtml

67 Yang J-H, Huang P-Y, Shie S-S, *et al.* Predictive Symptoms and Signs of Laboratory-confirmed Influenza: A Prospective Surveillance Study of Two Metropolitan Areas in Taiwan. *Medicine (Baltimore)* 2015;**94**:e1952. doi:10.1097/MD.0000000000001952

68 Aoki FY, Allen UD, Stiver HG, *et al.* The use of antiviral drugs for influenza: Guidance for practitioners 2012/2013. *The Canadian Journal of Infectious Diseases & Medical Microbiology* 2012;**23**:e79.

69 Centers for Disease Control and Prevention (CDC). Value of pharmacy-based influenza surveillance - Ontario, Canada, 2009. *MMWR Morb Mortal Wkly Rep* 2013;**62**:401–4.

70 Yoshida M, Matsui T, Ohkusa Y, *et al.* Seasonal influenza surveillance using prescription data for anti-influenza medications. *Jpn J Infect Dis* 2009;**62**:233–5.

71 Sugawara T, Ohkusa Y, Ibuka Y, *et al.* Real-time prescription surveillance and its application to monitoring seasonal influenza activity in Japan. *J Med Internet Res* 2012;**14**:e14. doi:10.2196/jmir.1881

72 Liu C-H, Wang J-L, Su C-P, *et al.* Oseltamivir use and outcomes during the 2009 influenza A H1N1 pandemic in Taiwan. *BMC Public Health* 2013;**13**:646. doi:10.1186/1471-2458-13-646

73 Havers F, Thaker S, Clippard JR, *et al.* Use of influenza antiviral agents by ambulatory care clinicians during the 2012-2013 influenza season. *Clin Infect Dis* 2014;**59**:774–82. doi:10.1093/cid/ciu422

74 Avadhanula V, Rodriguez CA, Devincenzo JP, *et al.* Respiratory viruses augment the adhesion of bacterial pathogens to respiratory epithelium in a viral species- and cell type-dependent manner. *J Virol* 2006;**80**:1629–36. doi:10.1128/JVI.80.4.1629-1636.2006

75 Tong HH, Blue LE, James MA, *et al.* Evaluation of the Virulence of aStreptococcus pneumoniae Neuraminidase-Deficient Mutant in Nasopharyngeal Colonization and Development of Otitis Media in the Chinchilla Model. *Infect Immun* 2000;**68**:921–4. doi:10.1128/IAI.68.2.921-924.2000

76 Delamaire M, Maugendre D, Moreno M, *et al.* Impaired Leucocyte Functions in Diabetic Patients. *Diabetic Medicine* 1997;**14**:29–34. doi:10.1002/(SICI)1096-9136(199701)14:1<29::AID-DIA300>3.0.CO;2-V

77 Sun L, Klein EY, Laxminarayan R. Seasonality and Temporal Correlation between Community Antibiotic Use and Resistance in the United States. *Clin Infect Dis* 2012;**55**:687–94. doi:10.1093/cid/cis509

78 Kwong JC, Maaten S, Upshur REG, *et al.* The effect of universal influenza immunization on antibiotic prescriptions: an ecological study. *Clin Infect Dis* 2009;**49**:750–6. doi:10.1086/605087

79 Glezen WP. Benefits of a Universal Influenza Immunization Program: More than the Reduction in the Use of Antibiotics. *Clin Infect Dis* 2009;**49**:757–8. doi:10.1086/605088

80 Simonsen L, Taylor RJ, Viboud C, *et al.* Mortality benefits of influenza vaccination in elderly people: an ongoing controversy. *Lancet Infect Dis* 2007;**7**:658–66. doi:10.1016/S1473-3099(07)70236-0

For peer review only

For peer review only - http://bmjopen.bmj.com/site/about/guidelines.xhtml

Table 1. Description of the 10 study years: date the vaccination campaign began, characteristics of the epidemic and control periods.

	Year	Campaign start date*	Epidemic period					Control period	
			Start	End	Duration	Peak**	Serotypes***	Start	End
1	2006/2007	12/10/06	2007w03	2007w09	7	2007w06	AH3N2	2007w18	2007w30
2	2007/2008	28/09/07	2008w02	2008w10	9	2008w06	AH1N1/B	2008w18	2008w30
3	2008/2009	10/10/08	2008w51	2009w08	10	2009w04	AH3N2	2009w18	2009w30
4	2009/2010	01/09/09	2009w37	2009w52	16	2009w49	AH1N1	2010w18	2010w30
5	2010/2011	24/09/10	2010w51	2011w07	9	2011w01	AH1N1	2011w18	2011w30
6	2011/2012	29/09/11	2012w05	2012w12	8	2012w08	AH3N2	2012w18	2012w30
7	2012/2013	28/09/12	2012w51	2013w11	13	2013w05	AH1N1/B	2013w18	2013w30
8	2013/2014	11/10/13	2014w05	2014w10	6	2014w07	AH1N1/AH3N2	2014w18	2014w30
9	2014/2015	16/10/14	2015s03	2015s11	9	2015s06	AH3N2	2015w18	2015w30
10	2015/20165	15/10/15	2016s04	2016s14	11	2016s11	B	2016w18	2016w30

* Established by an official communiqué of the national health insurance fund and the ministry of health

**Weeks of maximum incidence

***Serotypes: Dominant or co-dominant virus types and subtypes circulating during the season. This information is based on variable numbers of samples and uses virus detection techniques of different sensitivity. Moreover, several definitions of co-dominance may have been used, and this information is provided as a rough guide.

For peer review only - http://bmjopen.bmj.com/site/about/guidelines.xhtml

For peer review only

Table 2. List of ICD-10-CA codes included in the administrative case definition of influenza-like illness*.

Diagnosis*	ICD-10 code
Sinusitis	J01 or J32
Upper respiratory tract infection	J06.8 or J06.9
Influenza	J09 J10 or J11
Viral pneumonia	J12
Acute bronchitis or bronchitis NOS or obstructive bronchitis	J20 or J40 or J44.8
Bronchiolitis	J21
Acute lower respiratory tract infection, not otherwise specified	J22
COPD with acute lower respiratory tract infection (includes pneumonia)	J44.0
COPD with acute exacerbation	J441
Pleurisy	R09.1
Pneumonia	J13 or J14 or J15 or J16
Pneumonia, organism unspecified	J18

*Developed using pilot data from emergency departments in Edmonton, Alberta. ICD codes were extracted from randomly selected cases comprising 15% of all emergency department visits with a main ambulatory care diagnosis of influenza.

For peer review only

For peer review only - http://bmjopen.bmj.com/site/about/guidelines.xhtml

Table 3. Classification of diseases that can be covered by chronic disease status, in four risk categories, according to the recommendations of the high council for public health (HCSP) with the number of the corresponding condition or disease).

1-RESPIRATORY DISEASES
9 Severe forms of neurological or muscular conditions (including myopathy), severe epilepsy;
14 Severe chronic respiratory failure;
18 Cystic fibrosis;
20 Paraplegia;
25 Multiple sclerosis;
29 Active tuberculosis;
2-CARDIOVASCULAR DISEASES
1) Disabling stroke;
3 Chronic arterial disease with ischemic events;
5) Severe heart failure, serious arrhythmia, severe heart valve defects, severe congenital heart disease; severe congenital heart disease;
13 Coronary disease;
3-KIDNEY FAILURE
19) Severe chronic nephropathy and primary nephrotic syndrome;
4-NEOPLASMS and/or IMMUNE DEFICIENCIES
2 Bone marrow failure and other chronic cytopoenia;
6 Chronic active liver disease and cirrhosis;
7 severe primary immunodeficiency, requiring prolonged treatment, HIV infection;
21 Polyarteritis nodosa, acute disseminated lupus erythematosus, progressive generalised scleroderma;
22 Severe progressive rheumatoid arthritis;
28 Effects of organ transplantation;
30 Malignant tumours, malignant lymphatic or haematopoietic condition.

For peer review only

For peer review only - http://bmjopen.bmj.com/site/about/guidelines.xhtml

STROBE 2007 (v4) Statement—Checklist of items that should be included in reports of *case-control studies*

Section/Topic	Item #	Recommendation	Reported on page #
**Title and abstract**	1	(*a*) Indicate the study's design with a commonly used term in the title or the abstract	1
		(*b*) Provide in the abstract an informative and balanced summary of what was done and what was found	2
**Introduction**			
Background/rationale	2	Explain the scientific background and rationale for the investigation being reported	4
Objectives	3	State specific objectives, including any prespecified hypotheses	4
**Methods**			
Study design	4	Present key elements of study design early in the paper	5
Setting	5	Describe the setting, locations, and relevant dates, including periods of recruitment, exposure, follow-up, and data collection	5
Participants	6	(*a*) Give the eligibility criteria, and the sources and methods of case ascertainment and control selection. Give the rationale for the choice of cases and controls	6
		(*b*) For matched studies, give matching criteria and the number of controls per case	6
Variables	7	Clearly define all outcomes, exposures, predictors, potential confounders, and effect modifiers. Give diagnostic criteria, if applicable	8-10
Data sources/ measurement	8*	For each variable of interest, give sources of data and details of methods of assessment (measurement). Describe comparability of assessment methods if there is more than one group	8-10
Bias	9	Describe any efforts to address potential sources of bias	10-11
Study size	10	Explain how the study size was arrived at	Fixed study size
Quantitative variables	11	Explain how quantitative variables were handled in the analyses. If applicable, describe which groupings were chosen and why	8-10
Statistical methods	12	(*a*) Describe all statistical methods, including those used to control for confounding	7
		(*b*) Describe any methods used to examine subgroups and interactions	8
		(*c*) Explain how missing data were addressed	No missing data
		(*d*) If applicable, explain how matching of cases and controls was addressed	6
		(*e*) Describe any sensitivity analyses	6
**Results**			

For peer review only

Participants	13*	(a) Report numbers of individuals at each stage of study—eg numbers potentially eligible, examined for eligibility, confirmed eligible, included in the study, completing follow-up, and analysed	5
		(b) Give reasons for non-participation at each stage	No non-participant
		(c) Consider use of a flow diagram	After the analyzes
Descriptive data	14*	(a) Give characteristics of study participants (eg demographic, clinical, social) and information on exposures and potential confounders	After the analyses
		(b) Indicate number of participants with missing data for each variable of interest	After the analyses
Outcome data	15*	Report numbers in each exposure category, or summary measures of exposure	After the analyses
Main results	16	(*a*) Give unadjusted estimates and, if applicable, confounder-adjusted estimates and their precision (eg, 95% confidence interval). Make clear which confounders were adjusted for and why they were included	7
		(*b*) Report category boundaries when continuous variables were categorized	8-10
		(*c*) If relevant, consider translating estimates of relative risk into absolute risk for a meaningful time period	6
Other analyses	17	Report other analyses done—eg analyses of subgroups and interactions, and sensitivity analyses	6
**Discussion**			
Key results	18	Summarise key results with reference to study objectives	No
Limitations	19	Discuss limitations of the study, taking into account sources of potential bias or imprecision. Discuss both direction and magnitude of any potential bias	10-11
Interpretation	20	Give a cautious overall interpretation of results considering objectives, limitations, multiplicity of analyses, results from similar studies, and other relevant evidence	15
Generalisability	21	Discuss the generalisability (external validity) of the study results	No
**Other information**			
Funding	22	Give the source of funding and the role of the funders for the present study and, if applicable, for the original study on which the present article is based	15

*Give information separately for cases and controls in case-control studies and, if applicable, for exposed and unexposed groups in cohort and cross-sectional studies.

For peer review only

Note: An Explanation and Elaboration article discusses each checklist item and gives methodological background and published examples of transparent reporting. The STROBE checklist is best used in conjunction with this article (freely available on the Web sites of PLoS Medicine at http://www.plosmedicine.org/, Annals of Internal Medicine at http://www.annals.org/, and Epidemiology at http://www.epidem.com/). Information on the STROBE Initiative is available at www.strobe-statement.org.

For peer review only
